# Long-chain polyphosphates induce glomerular microthrombi and exacerbate LPS-induced acute kidney injury in mouse

**DOI:** 10.1242/dmm.052361

**Published:** 2026-03-30

**Authors:** Anniina Pirttiniemi, Hanne Salmenkari, Krishna Adeshara, Jere Lindén, Sanna Lehtonen, Niina Sandholm, Per-Henrik Groop, Markku Lehto

**Affiliations:** ^1^ Folkhälsan Institute of Genetics, Folkhälsan Research Center, 00290 Helsinki, Finland; ^2^ Department of Nephrology, University of Helsinki and Helsinki University Hospital, 00290 Helsinki, Finland; ^3^ Program for Clinical and Molecular Metabolism, Faculty of Medicine, University of Helsinki, 00290 Helsinki, Finland; ^4^ Department of Veterinary Biosciences, University of Helsinki, 00014 Helsinki, Finland; ^5^ Finnish Centre for Laboratory Animal Pathology, HiLIFE, University of Helsinki, 00014 Helsinki, Finland; ^6^ Department of Pathology, University of Helsinki, 00014 Helsinki, Finland; ^7^ Department of Diabetes, Central Clinical School, Monash University, Melbourne, VIC 3004, Australia; ^8^ Baker Heart and Diabetes Institute, Melbourne, VIC 3004, Australia

**Keywords:** Polyphosphates (PolyPs), Lipopolysaccharide, Acute kidney injury, Glomerular thrombosis, Podocyte injury, Cytokines

## Abstract

Polyphosphates (PolyPs) are evolutionarily conserved anionic polymers mediating pleiotropic functions in eukaryotes and prokaryotes, depending on their chain length. Bacteria typically synthetize long chains, while human platelets harbor exclusively medium chains. PolyP-mediated lung and liver-injury have been reported in experimental mouse models but their effects on the kidney remain undefined. Here, we assessed kidney histopathology and cytokine levels following intravenous administration of medium-chain (P100) and long-chain (P700) PolyPs and their synergistic effects with lipopolysaccharides (LPS) in mice. We found that P700 induced albuminuria, renal transcription of *Kim-1* and *Lcn2*, focal renal damage with glomerular microthrombi, tubular degeneration, granular phenotype of slit diaphragm components nephrin and ZO1 (also known as TJP1), and enlarged electron-dense vesicles in podocyte cytoplasm indicating lysosome swelling. P700 combined with LPS induced marked multifocal acute tubular necrosis in the cortex and augmented LPS-induced pro-inflammatory cytokine levels. No notable effects were seen with P100, indicating that development of PolyP-mediated kidney injury is dependent on chain length. We conclude that P700 PolyPs may play a procoagulant role in kidney injury development, by inducing microthrombi characteristic of thrombotic microangiopathy and augmenting cytokine levels under inflammatory conditions.

## Teaser

**Summary:** Intravenous administration of long-chain polyphosphates induces renal damage with glomerular microthrombi and augments lipopolysaccharide-induced cytokine levels in mice, indicating a procoagulant and cytokine amplifying role for polyphosphates under inflammatory conditions.

## INTRODUCTION

Polyphosphates (PolyPs) are evolutionarily conserved linear phosphate polymers, which have been studied increasingly in recent years for their ability to regulate various biological processes (Schoeppe et al., 2024). They are synthetized ubiquitously in eukaryotes and prokaryotes. Longer chains of 100 to 1000 phosphate residues are synthetized in bacterial cells, where they regulate cellular energy metabolism and virulence (Achbergerová and Nahálka, 2011; Müller et al., 2009). Upon lysis of pathogenic gram-negative bacteria, PolyPs may be released together with pathogen-associated molecular patterns (PAMPs), such as lipopolysaccharides (LPSs) (Shah et al., 2024; Rashid et al., 2000). In rodent tissues PolyPs have been identified in several organelles, e.g. in lysosomes, mitochondria, dense granules and nuclei (Pisoni and Lindley, 1992; Müller et al., 2019; Kornberg, 1995), with chain lengths ranging from 50 to 800 phosphate residues and concentrations ranging from ∼25 to 120 μM (Kumble and Kornberg, 1995). Particularly high concentrations of ≤130 mM of medium-chain PolyPs (∼50-75 phosphate residues) are stored in platelets and mast cells, from where they can be secreted upon activation (Docampo and Moreno, 2011; Huang et al., 2023; Ruiz et al., 2004).

PolyPs have pleiotropic impact on cellular function, and previous studies have shown variable outcomes depending on their chain length, dose, and route of administration. Exogenously administered PolyPs have been reported to improve wound healing (Müller et al., 2017; Schepler et al., 2022), intestinal health (Fujiya et al., 2019; Isozaki et al., 2021; Takauji et al., 2021) and bone mineralization (Wang et al., 2012), while endogenous PolyP accumulation in the brain has been associated with neurodegenerative diseases, such as amyotrophic lateral sclerosis and frontotemporal dementia (Garcés et al., 2024). PolyPs modulate systemic inflammation in a chain length-dependent manner, i.e. long-chain PolyPs of ∼700 phosphate residues, can inhibit type I interferon signaling in immune cells, and augment *E. coli*/LPS-induced pro-inflammatory cytokine release and mortality, while, conversely, shorter PolyPs (∼150 phosphate residues administered intravenously) have shown protective effects against mortality in mice (Pirttiniemi et al., 2023; Roewe et al., 2020; Terashima-Hasegawa et al., 2019; Ito et al., 2020). Previous studies have demonstrated that high doses of PolyPs administered intravenously, intraperitoneally or intratracheally can induce acute lung and liver injury in mice, mediated by immunomodulation and coagulation (Müller et al., 2009; Terashima-Hasegawa et al., 2019; Roewe et al., 2022; Krenzlin et al., 2023; Zilberman-Rudenko et al., 2018); however the impact of PolyPs on the kidneys has not been assessed.

Acute kidney injury (AKI) is a major cause of morbidity and mortality worldwide, and can predispose to conditions, such as multi-organ failure and chronic kidney disease. Infection-induced renal injury, such as AKI, may be directly mediated by microorganisms or indirectly mediated by immune system activation. Sepsis-induced AKI is common among the severe cases and is characterized by kidney inflammation, cytokine release, and microcirculatory alterations causing hypoperfusion and hypoxia (Turgut et al., 2023). AKI has been experimentally modelled in mice, most commonly with ischemia–reperfusion injury, exposure to nephrotoxic agents, and in models of septic AKI, with cecal ligation puncture or endotoxemia (Liang and Liu, 2023; Manrique-Caballero et al., 2021).

Here, our aim was to investigate the impact of PolyP exposure on the kidney by assessing kidney injury markers, histopathology and cytokine levels in mice. Since different lengths of PolyPs may be released to circulation, e.g. from platelets or bacteria (Schoeppe et al., 2024; Rashid et al., 2000; Ruiz et al., 2004), we studied the effects of intravenously administered medium-chain (P100) and long-chain (P700) PolyPs, as well as their impact on LPS-induced AKI phenotype. We hypothesized that PolyPs can affect kidney function or alter LPS-induced kidney injury development in a chain length-dependent manner.

## RESULTS

### P700 induces albuminuria and transcriptional markers of kidney injury, and augments LPS-induced cytokine levels

P100 or P700 treatment induced no clinical signs of sickness in mice during our pilot experiment (3-day follow up, *n=*2) or the final experiment (20-h follow up, *n=*4), whereas LPS-treated mice exhibited typical clinical signs of sepsis within 20 h in the pilot (*n=*2) and in the final experiment (*n=*8), i.e. hunched posture, piloerection and dehydration. In the final experiment P100 did not alter the LPS-induced clinical signs (*n=*8), whereas P700 worsened them as mice in the LPS+P700 group deteriorated rapidly, with two out of eight mice of that group dying prior to sample collection at 20 h post treatment.

**Fig. 1.**
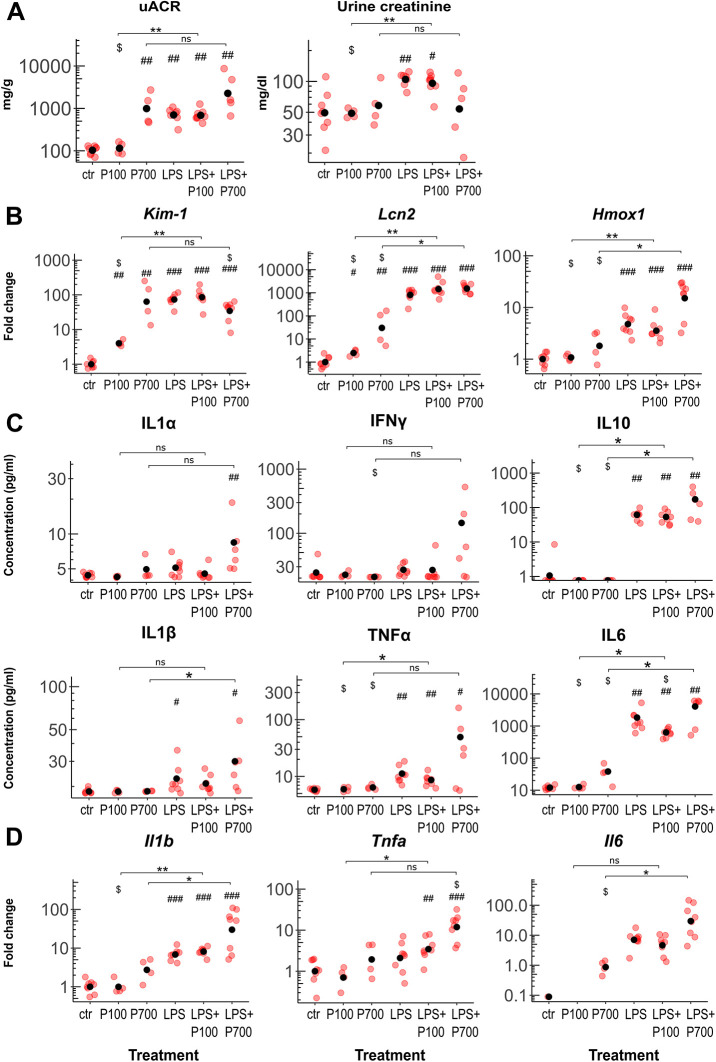
**Markers of acute kidney injury in PolyP- and/or LPS-treated mice.** Mice were treated with medium-chain (P100) or long-chain (P700) PolyPs alone, or in combination with LPS (LPS+P100 or LPS+P700, respectively) (*n*=4-8 mice per group). (A) Urine albumin:creatinine ratio (uACR) and urine creatinine levels of the treated mice. (B) Transcriptional levels of kidney injury markers *Kim-1* and *Lcn2,* and *Hmox1*, measured by RT-qPCR from the kidney tissue. (C) Levels of serum cytokines (IL1α, IFNγ, IL10, IL1β, TNFα, IL6) in treated mice measured by a multiplex ELISA assay. (D) Transcriptional levels of cytokines (*IL1b*, *Tnfa*, *Il6*) measured by RT-qPCR from the kidney tissue. For plots in A and C, red dots indicate the concentration values of each mouse, black dots indicate the arithmetic means per group. For plots in B and D, red dots indicate the relative gene expression of each mouse compared to control group, black dots indicate the geometric mean per group, scaled as one in the control group. For plotted levels of *Il6*, the P700 group was scaled as one. Benjamini-Hochberg adjusted significance levels were calculated using the pairwise Mann–Whitney *U*-test (significance levels: one symbol *P*<0.05, two symbols *P*<0.01, three symbols *P*<0.001). Symbols used for significance: #, significance compared to the control group; $, significance compared to the LPS-treated group; ns, non-significant.

Kidney function was assessed 20 h post treatment in the final experiment by assessing the urine albumin:creatinine ratio (uACR). While P100 treatment had no significant effects, P700 elevated the uACR significantly, to levels similar to those observed after LPS treatment, when compared to untreated controls. The highest uACR was seen in the LPS+P700 group. Of note, neither P100 nor P700 did elevate the level of urine creatinine, as was seen in the LPS-treated groups (Fig. 1A).

Transcription of kidney injury markers hepatitis A virus cellular receptor 1 [*Havcr1*; also known as kidney injury molecule 1 (*Kim-1*)], lipocalin 2 (*Lcn2*) and heme oxygenase 1 (*Hmox1*) was analyzed by using reverse transcription–quantitative polymerase chain reaction (RT-qPCR). Treatment with P100 caused a minor elevation to the transcription levels of *Kim-1* and *Lcn2,* whereas P700 treatment elevated transcription of all injury markers more markedly. LPS significantly elevated transcription of all injury markers, and P700 treatment moderately lowered the LPS-induced transcription of *Kim-1* but elevated the LPS-induced transcription of *Hmox1* (Fig. 1B).

Levels of cytokines (IL1α, IFNγ, IL10, IL1β, TNFα, IL6) were measured in the serum by using multiplex ELISA (Fig. 1C) and in kidney tissue by using RT-qPCR (Fig. 1D). While treatment with P100 or P700 did not cause significant cytokine elevation compared to that of controls, LPS caused significant elevation in serum levels of IL10, IL1β, TNFα and IL6 and in transcription levels of *Il1b* in kidney tissue. The LPS-induced serum levels of IL6 were slightly decreased in response to P100, whereas P700 caused an increasing trend in all LPS-induced cytokine levels in serum and kidney tissue (Fig. 1C,D).

We assessed the transcription of the interferon stimulated genes *Ifit2*, *Isg15* and *Oas1a*, which are downregulated by P700 in human and murine leukocytes (Pirttiniemi et al., 2023; Roewe et al., 2020). Conversely, in kidney tissue, P100 or P700 did not notably affect transcription of these genes, but P700 augmented the LPS-induced transcription of *Ifit2* and *Isg15* (Fig. S1A).

### P700 induces focal tubular inflammation, apoptosis and formation of protein casts in the kidney, and marked acute tubular necrosis when combined with LPS

**Fig. 2.**
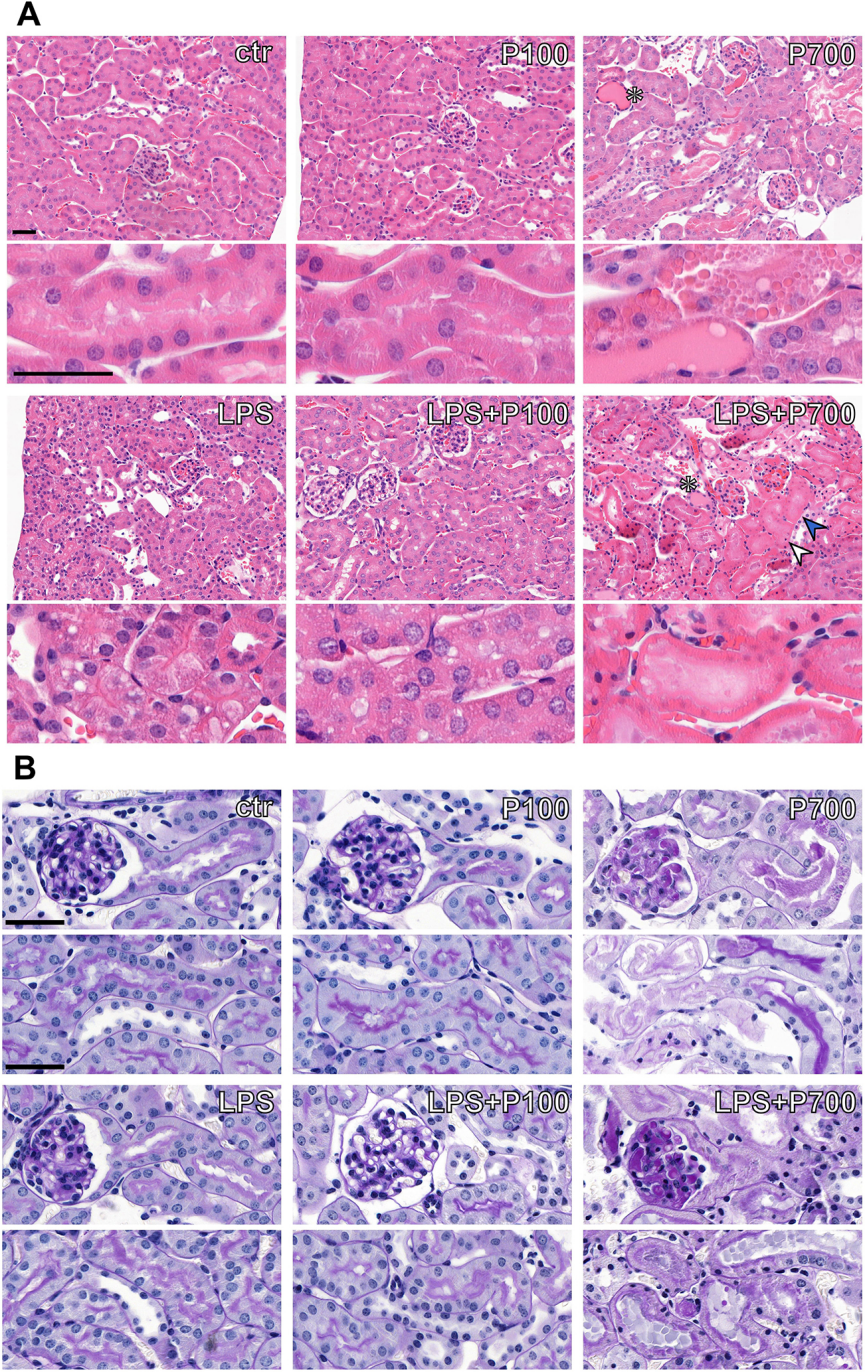
**Histopathological manifestations of acute kidney injury seen in PolyP and/or LPS-treated mice.** (A) H&E staining showing kidney histopathological changes seen in mice treated with medium-chain (P100) or long-chain (P700) PolyPs alone, or in combination with LPS (LPS+P100 or LPS+P700, respectively). P700-treated mice show foci of acute tubular necrosis and tubular casts most prominent in the outer medulla (white asterisks). LPS+P700-treated mice additionally exhibit extensive acute tubular necrosis in the cortex, manifested as cytoplasmic eosinophilia, pyknotic nuclei (white arrowhead), karyolysis (blue arrowhead) and tubular casts. LPS- or LPS+P100-treated mice display minimal tubular degeneration, with mildly swollen and vacuolated cytoplasm. (B) PAS-staining shows glomerular congestion with deposits in the capillary loops, as well as loss of adjacent proximal tubule brush border integrity in affected foci of P700 and LPS+P700-groups. Images extracted from a 40× whole-slide scan. All scale bars: 40 µm.

Histopathological signs of kidney injury were assessed with hematoxylin & eosin (H&E) and Periodic acid–Schiff (PAS)-stained kidney sections (Fig. 2A,B; Table S1). While P100 treatment caused no histological alterations, P700-treated mice displayed foci of tubular degeneration as well as acute tubular necrosis in the cortex and outer stripe of the outer medulla. The degenerated tubular segments exhibited granular eosinophilic cytoplasm and the necrotic segments, nuclear pyknosis, karyorrhexis or karyolysis and cell sloughing. Additionally, protein casts were present in the collecting ducts or tubules (*pars recta*) and the collecting ducts in inner medulla (Fig. 2A, Table S1). Glomerular congestion and disruption of adjacent proximal tubule brush borders were observed focally in P700-treated mice (Fig. 2B). LPS-treated mice typically displayed minimal tubular degeneration in multiple foci within the subcapsular cortex, with epithelial cells showing mild swelling, increased eosinophilia and vacuolated cytoplasm (Fig. 2A). P100 did not modify the LPS-induced histological changes except from occasional dilated glomerular capillaries (Fig. 2A,B). The histological lesions in the LPS+P700-treated group were similar to those treated with P700 or LPS individually but, additionally, exhibited marked multifocal acute tubular necrosis (Fig. 2A,B; Table S1). Acute phosphate poisoning/nephrotoxicity (Fogo et al., 2017) was ruled out by von Kossa staining, which showed no signs of calcium deposits in the kidney (Fig. S1B).

**Fig. 3.**
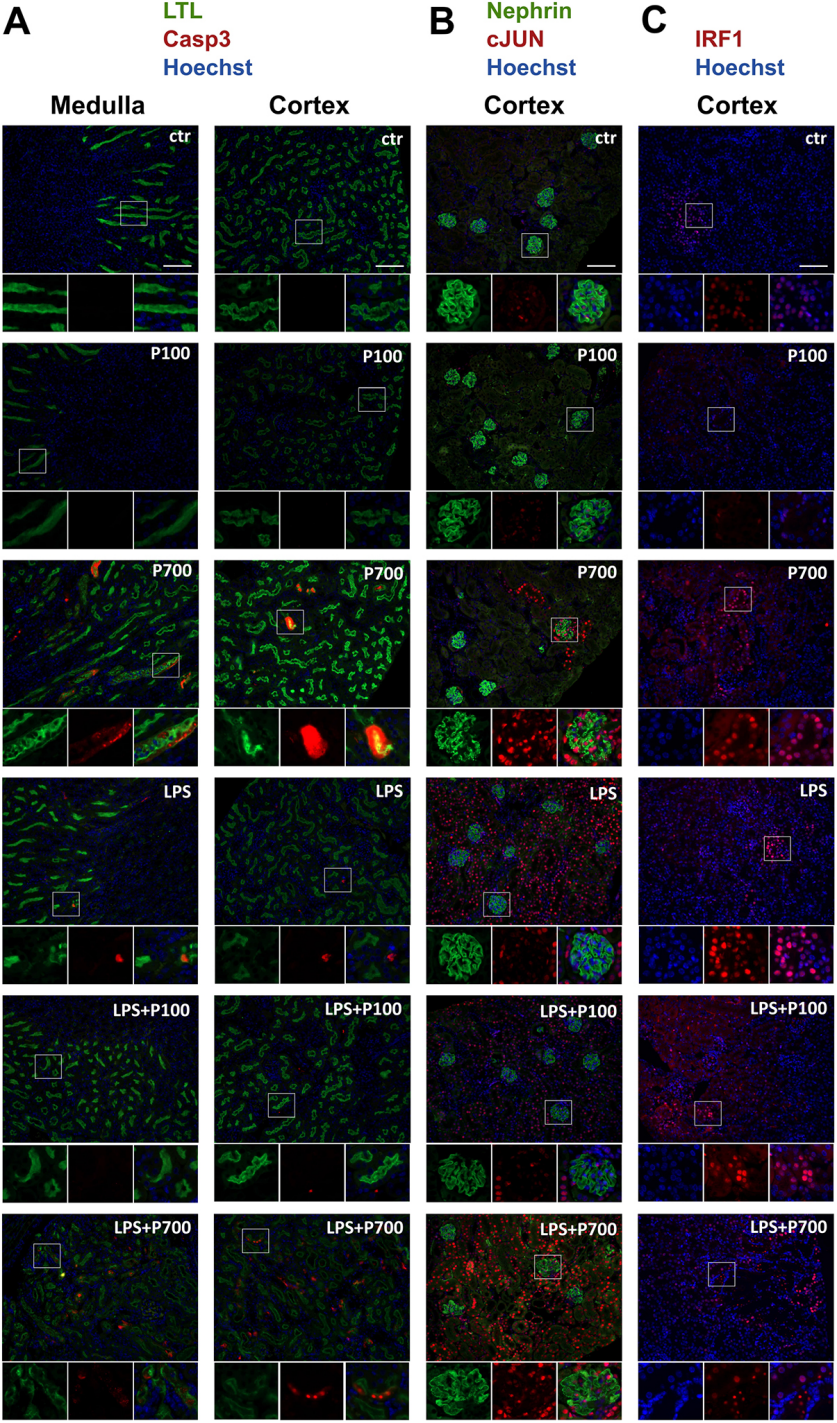
**Kidney tubular apoptosis and expression of the inflammatory markers cJUN and IRF1 in PolyP and/or LPS-treated mice.** Mice were treated with medium-chain (P100) or long-chain (P700) PolyPs alone, or in combination with LPS (LPS+P100 or LPS+P700, respectively). (A) Immunofluorescence staining of apoptotic cells against cleaved caspase 3 (Casp3, red) and of kidney proximal tubule brush borders with *lotus tetragonolobus* lectin (LTL, green). Apoptosis inside disrupted proximal tubules was seen in mice treated with P700, most prominently in the outer medulla. In LPS+P700-treated mice several individual apoptotic tubular and glomerular cells were additionally seen in affected foci within the cortex. Only few apoptotic cells were seen in LPS-and LPS+P100-treated groups. (B) The kidney cortex showed distinct cJUN-positive (red) foci in P700-treated mice, which were located adjacent to the glomeruli with a granular nephrin (green) phenotype. Mice treated with LPS, LPS+P100 or LPS+P700 showed evenly distributed cJUN protein expression throughout the cortex, independent of the glomeruli with a granular nephrin phenotype. (C) Foci with IRF1-positive (red) nuclei were slightly increased in the kidney cortex in response to treatment with P700 or LPS compared to controls. Strong IRF1-expression was seen in pyknotic nuclei in necrotic areas of LPS+P700-treated mice. Images were acquired using a 20× objective. Boxed areas below each main image show individual staining (left, middle) and merged images (right). Nuclei were stained with Hoechst 33258 (blue). All scale bars: 100 µm.

Proximal tubule brush border morphology and tubular apoptosis were further assessed by immunostaining with *lotus tetragonolobus* lectin (LTL) and against cleaved caspase 3, respectively (Fig. 3A). No changes were seen in the P100-group compared to controls, whereas the P700-group showed loss of proximal tubule brush border integrity and tubular apoptosis within proximal tubuli, focally in the outer medulla. LPS-treated and LPS+P100-treated mice showed only few apoptotic cells in the medulla and cortex. The LPS+P700-treated group displayed similar apoptotic proximal tubuli as P700-treated group in the medulla but, additionally, exhibited tubular and glomerular cells in active apoptosis in the cortex (Fig. 3A).

Immunostaining for the Jun proto-oncogene (c-Jun, hereafter referred to as cJUN), a marker associated with several kidney diseases (De Borst et al., 2007; Smith et al., 2021) showed only few weakly stained nuclei in glomerular and tubular cells of control and P100-treated mice. All P700-treated mice displayed distinct isolated foci with marked glomerular and tubular cJUN-positive nuclei in the cortex, whereas all mice in the LPS-treated groups displayed diffusely distributed tubular cJUN expression in the cortex (Fig. 3B). Staining for the pro-inflammatory transcription factor IRF1 (Wang et al., 2009) showed cortical foci with tubular nuclear expression in all groups. Although the number of IRF1-positive foci were only slightly increased in the P700- and LPS-treated groups compared to controls, notably strong nuclear IRF1-expression was seen in the apoptotic/necrotic foci in the LPS+P700 group, especially in the pyknotic nuclei, which no longer stain positive for Hoechst 33258 (Fig. 3C).

Leukocyte infiltration in kidney tissue was assessed with immunostaining of the tissue-resident macrophage marker protein F4/80. While P100 did not cause a general increase in the F4/80-positive area compared to that of controls, all other treatment groups showed an increasing trend of macrophage-covered area. Areas most covered in macrophages were seen in the LPS+P700-treated mice, particularly in the most damaged foci (Fig. S2A,B). Moreover, glomerular intracapillary leukocytes were only seen in the LPS-treated groups when using transmission electron microscopy (TEM) analysis (Fig. S2C; Table S2).

### P700-affected foci in the kidney display glomerular microthrombi comprising adhered platelets, von Willebrand factor, fibrin and collagen fibrils

**Fig. 4.**
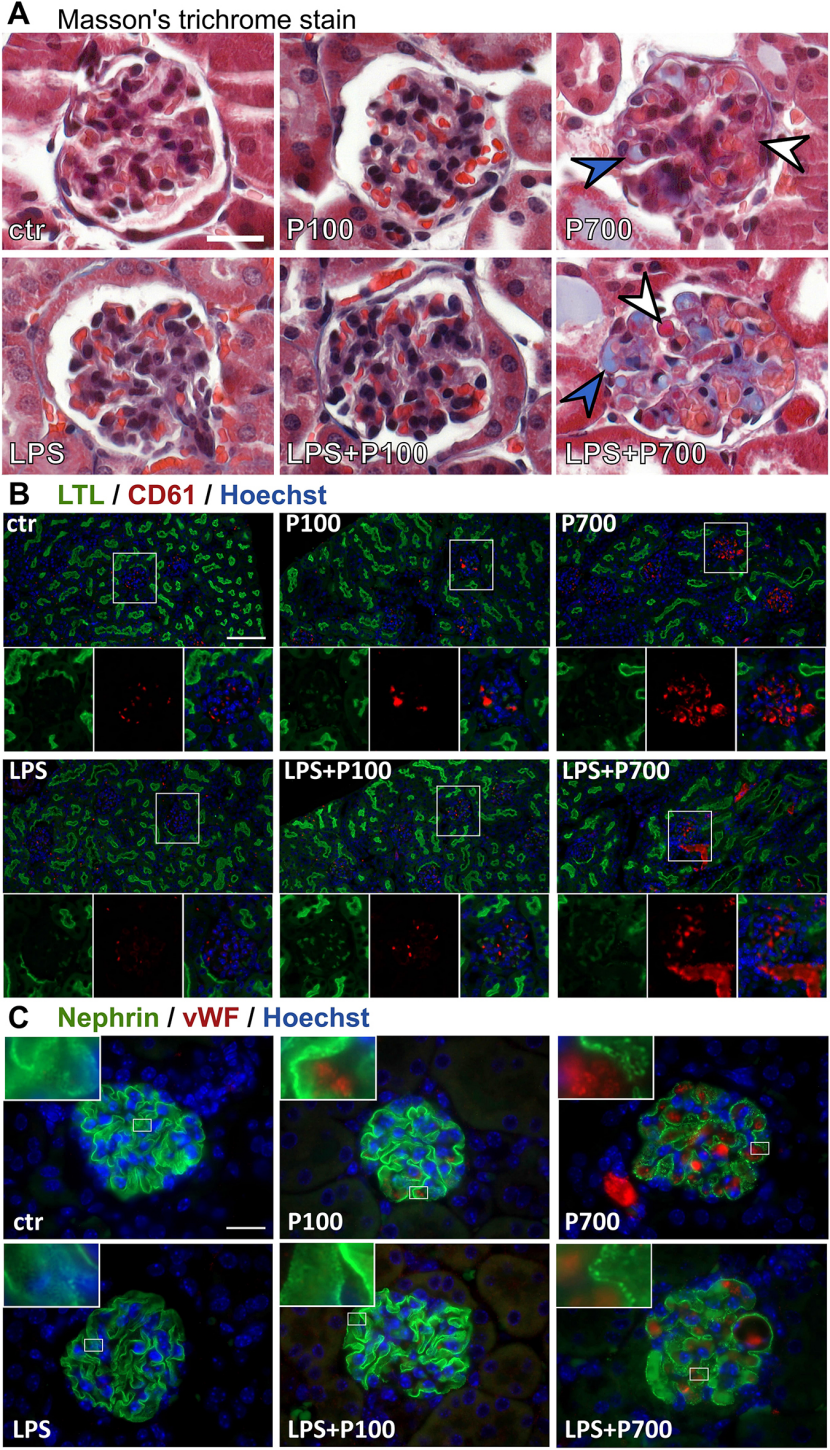
**Glomerular microthrombi consisting of fibrin/collagen, von Willebrand factor and platelet accumulation in the kidney cortex of PolyP and/or LPS-treated mice.** Mice were treated with medium-chain (P100) or long-chain (P700) PolyPs alone, or in combination with LPS (LPS+P100 or LPS+P700, respectively). (A) Masson's trichrome stain shows red-stained deposits indicating fibrin (white arrowheads) and blue-stained deposits indicating collagen (blue arrowheads) in the glomerular capillary loops of P700- and LPS+P700-treated mice. Images extracted from a 40× whole-slide scan. Scale bar: 20 µm. (B) Immunofluorescence staining of platelets with antibody against CD61 (red) and of kidney proximal tubule brush borders with *lotus tetragonolobus* lectin (LTL, green) show platelet accumulation in glomeruli and arterioles of P700- and LPS+P700-treated mice. Images were acquired using a 20× objective. Boxed areas below each main image show individual staining (left, middle) and merged images (right). Nuclei were stained with Hoechst 33258 (blue). Scale bar: 100 µm. (C) Immunofluorescence staining showing von Willebrand factor-positive (vWF, red) deposits in the glomerular capillary loops of P700 and LPS+P700-treated mice, with the podocyte marker, nephrin (green) showing a granular phenotype. Images were acquired using a 100× objective. Magnified views are shown in the top left of each image. Nuclei were stained with Hoechst 33258 (blue). Scale bar: 20 µm.

The P700-induced glomerular congestion was assessed further with Masson's trichrome staining, which revealed intracapillary deposits indicative of fibrin (stained red) and collagen (stained blue), as well as erythrocyte accumulation (Fig. 4A, Table S1). Collagen fragments may be released to circulation by matrix metalloproteinase-mediated degradation from sources like exposed endothelial or glomerular basement membrane and participate in early thrombus formation with fibrin (Sand et al., 2013; Manon-Jensen et al., 2016). These signs of thrombotic events were further supported by immunostaining for the platelet-marker CD61 and the inductor of platelet adhesion von Willebrand factor (vWF), which revealed conspicuous glomerular microthrombi. Several glomeruli in the P700-treated group displayed CD61-positive platelet accumulation in the capillary loops and the adjacent arterioles (Fig. 4B), and notable intracapillary vWF-positive deposits (Fig. 4C). The P100-treated mice showed no signs of thrombosis, apart from a few glomeruli with slight increase of CD61 and vWF protein expression (Fig. 4A-C; Table S1). The dose of LPS used here did not induce glomerular thrombosis. LPS+P700-treated mice showed similar indicators of glomerular thrombosis as P700-treated mice but showed more pronounced collagen deposition (blue) with Masson's trichrome staining (Fig. 4A-C; Table S1).

**Fig. 5.**
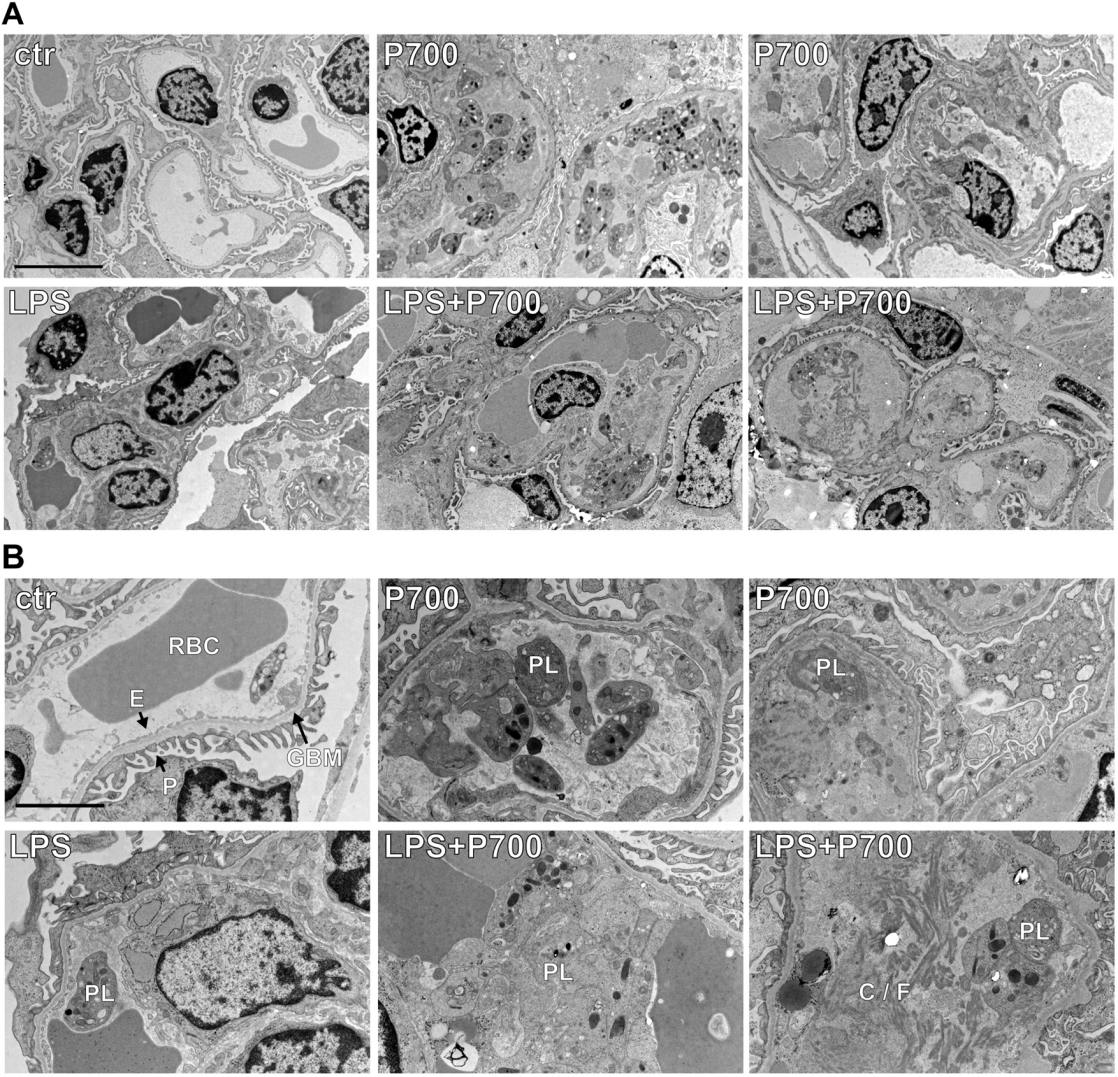
**Transmission electron microscopy imaging of glomerular microthrombi in the kidney cortex of mice treated with long-chain PolyPs and/or LPS.** (A,B) Transmission electron microscopy images showing glomerular microthrombi consisting of adhered platelets and cellular debris in mice treated with long-chain (P700) PolyPs alone. Mice treated with P700 in combination with LPS (LPS+P700) additionally display structures indicative of collagen fibrils and/or fibrin strands. Individual non-adhered intracapillary platelets can be seen in control and LPS-treated mice. Magnification: 1200× (A), 3000× (B). Scale bars: 5 µm (A), 2 µm (B). E, fenestrated endothelium; P, podocyte foot processes; GBM, glomerular basement membrane; RBC, red blood cell; PL, platelets; C/F, collagen fibrils/fibrin strands.

Thrombic clot composition was confirmed for the P700- and LPS-treated groups by TEM. Adhered platelets and cellular debris were seen in the capillary loops of the affected glomeruli in the P700-treated mice. LPS-treated mice showed only individual non-adhered intracapillary platelets, similar to controls. The affected glomeruli in the LPS+P700-group displayed adhered platelets and debris and, in one mouse, structures indicative of collagen fibrils and fibrin strands were additionally detected (Fig. 5A,B; Table S2).

### P700-affected glomeruli display mislocalization of slit diaphragm proteins and enlarged lysosomes in cytoplasm of podocytes

**Fig. 6.**
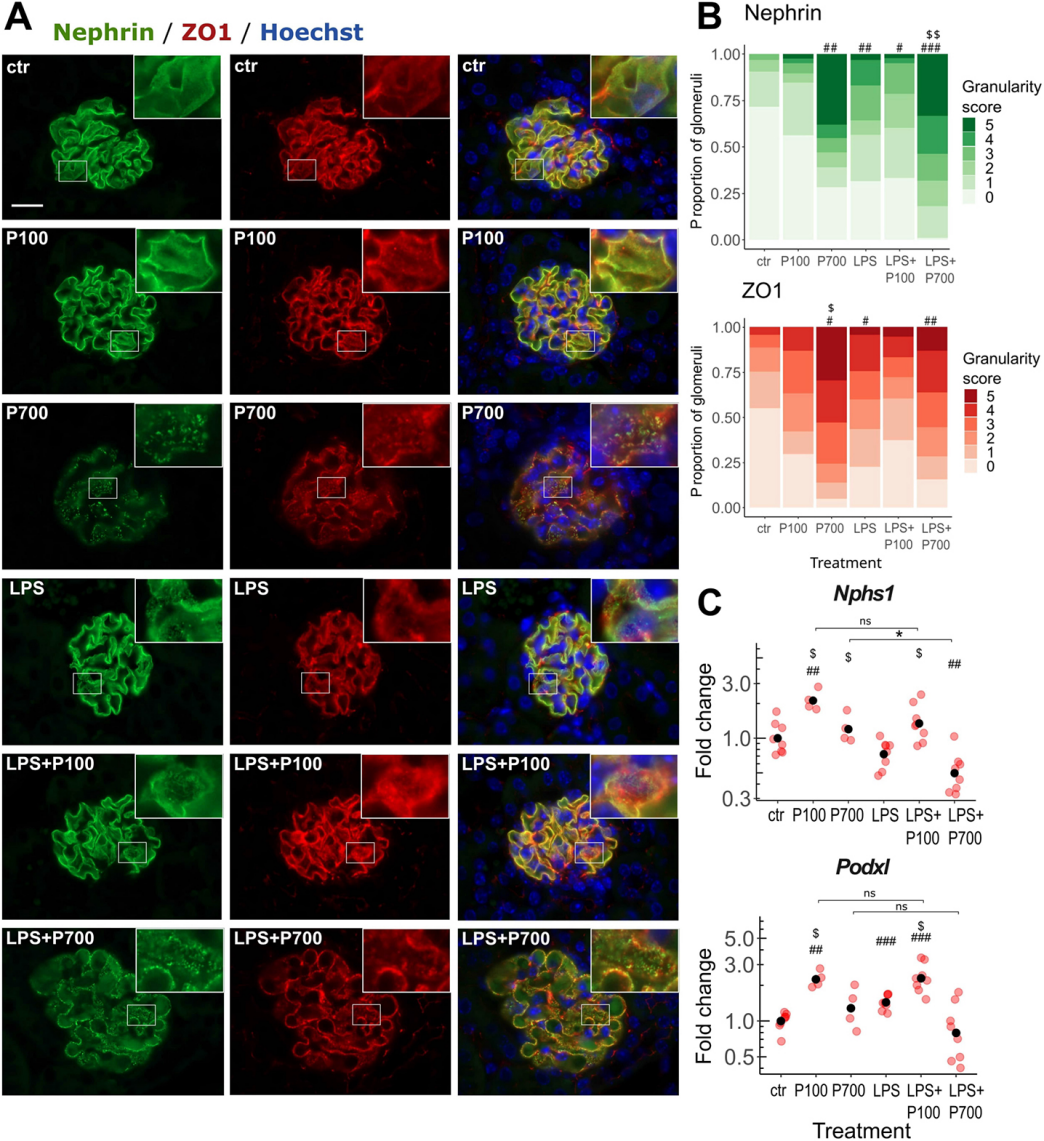
**P700-induced granular phenotype of slit diaphragm components, nephrin and ZO1.** (A) Representative images of glomeruli, immunostained with the podocyte-marker nephrin (green) and the tight junction-marker ZO1 (red) in mice treated with medium-chain (P100) or long-chain (P700) PolyPs alone, or in combination with LPS (LPS+P100 or LPS+P700, respectively). Boxed areas are shown magnified at top right of each image. Nuclei were stained with Hoechst 33258 (blue). Images were acquired using a 100× objective. Scale bar: 20 µm. (B) Proportions and degree of granularity of the nephrin and ZO1-imaged glomeruli. Stacked bars represent the treatment group average of mouse granularity score proportions. Significance levels between the groups are calculated using a granularity score mean value for each mouse (*n*=4-8 mice per group, 7-27 imaged glomeruli per mouse). (C) Transcription levels of *Nphs1* and *Podxl* in kidney tissue obtained from mice as indicated measured by RT-qPCR. Red dots indicate the relative gene expression of each mouse compared to the control group. Black dots indicate the geometric mean per group, scaled as one in the control group. Benjamini-Hochberg-adjusted significance levels indicated in B and C were calculated using the pairwise Mann–Whitney *U*-test (significance levels: one symbol *P*<0.05, two symbols *P*<0.01, three symbols *P*<0.001). Symbols used for significance: #, significance compared to the control group; $, significance compared to the LPS-treated group. ns, non-significant.

Glomerular slit diaphragm composition was assessed by immunostaining for the transmembrane podocyte marker nephrin (NPHS1) and tight junction protein 1 (TJP1, also known as, and hereafter referred to, as ZO1), which showed an evenly distributed expression of both proteins, lining the glomerular capillary loops in controls (Fig. 6A). A distinct granular phenotype for both proteins, with more distinguishable foot processes, was observed, particularly, in the P700-treated mice, and a granularity score (ranging from 0 to 5) was used to assess the degree of granularity in all groups (Fig. 6B; Fig. S3A). While P100 caused only a slight increase in the granularity score, P700 treatment induced notable granularity in several glomeruli. LPS induced less but still significant levels of granularity. The LPS+P700 group showed the highest proportion of overall granularity for nephrin, while the P700 group showed the highest overall granularity for ZO1 (Fig. 6A,B; Fig. S3A). Nevertheless, the glomerular nephrin and ZO1 granularity scores correlated with each other in the P700, LPS and LPS+P700 groups and when all groups were combined (Fig. S3B). Of note, the granularity was global, affecting the entire glomerulus, although the granular glomeruli were focally distributed in the cortex in all affected groups. The transcriptional levels of nephrin (*Nphs1*) and the podocyte glycocalyx component podocalyxin (*Podxl*) showed a small increase in the P100 and LPS+P100 groups and a small decrease in the LPS+P700 group (Fig. 6C).

The connection between slit diaphragm integrity loss and glomerular microthrombi was investigated by comparing the glomerular nephrin granularity score with the vWF-positive area per frame. The most significant correlation was seen in the P700 and LPS+P700 groups, but also in the P100 group, the controls and when all groups were combined. Interestingly, no correlation was seen in the LPS and LPS+P100 groups. (Fig. 4C; Fig. S4A-C). Moreover, in P700-treated mice glomeruli displaying notable nephrin granularity were generally overlapping with or located adjacent to the foci of cJUN-positive tubuli (Fig. 3B).

**Fig. 7.**
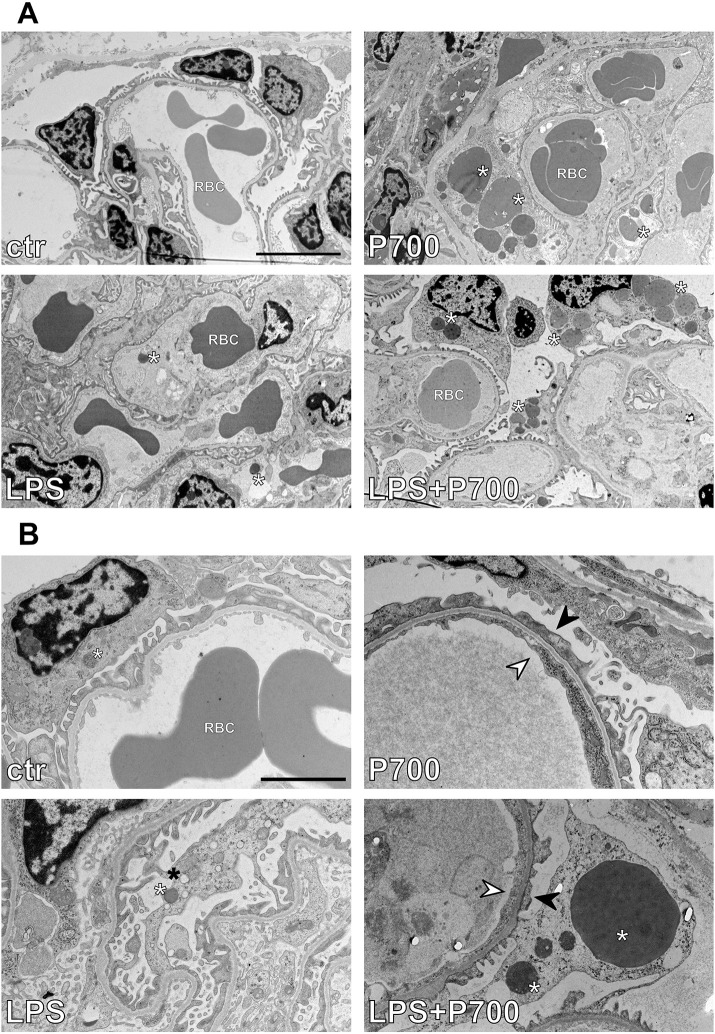
**Lysosome swelling and ultrastructural changes in glomerular podocytes and endothelial cells in the mice treated with long-chain PolyPs and/or LPS.** (A,B) Transmission electron microscopy images of glomerular podocytes and endothelial cells in mice treated with long-chain (P700) PolyPs alone or in combination with LPS (LPS+P700), showing large podocytic electron-dense vesicles characteristic of lysosomes (white asterisks) in both P700- as well as LPS+P700-treated mice. (B) Loss of endothelial fenestration (white arrowheads) and podocyte foot process morphology (black arrowheads) was also seen in the most severely affected glomeruli. Some podocytic and endothelial lysosome enlargement as well as vacuolization (black asterisk) was seen in the most affected glomeruli in LPS-treated mice. Magnification: 1200× (A), 3000× (B). Scale bars: 5 µm (A), 2 µm (B). RBC, red blood cell.

Glomerular podocyte and endothelial cell ultrastructural changes were analyzed with TEM in the P700- and LPS-treated groups. P700-treated mice showed enlarged electron-dense vesicles, characteristic of lysosomes in podocyte cytoplasm of the affected glomeruli. While enlarged podocytic vesicles/lysosomes were not seen in LPS-treated mice, LPS+P700-treated mice showed the most notable increase in podocytic vesicle size (Fig. 7A,B; Fig. S5). In glomerular endothelial cells, P700 did not cause an increase in cytoplasmic vesicle size; however, some increase was seen in the LPS and LPS+P700 groups (Fig. 7A,B; Fig. S6). The most severely affected glomerular segments in P700-, LPS- or LPS+P700-treated mice showed also loss of morphology, e.g. podocyte vacuolization, foot process fusion and loss of endothelial fenestration (Fig. 7A,B; Table S2).

### P700 causes focal alterations in tubular kallikrein–kinin system components and augments LPS-induced transcription of tissue factor-encoding *F3*

**Fig. 8.**
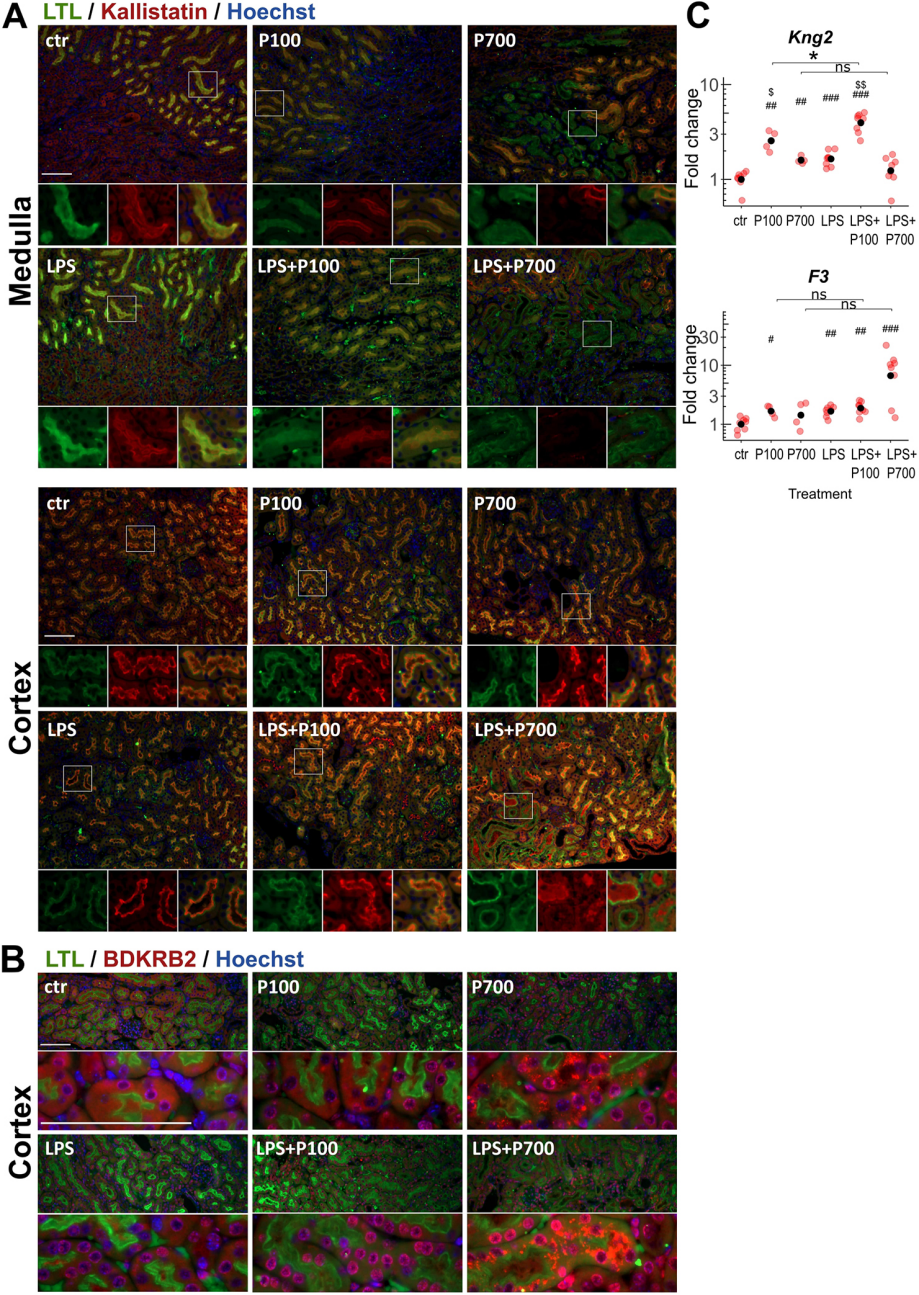
**Expression of kallikrein–kinin-system components and tissue factor in kidney of PolyP and/or LPS-treated mice.** Mice were treated with medium-chain (P100) or long-chain (P700) PolyPs alone, or in combination with LPS (LPS+P100 or LPS+P700, respectively) (A) Top: Immunofluorescence staining for the tissue kallikrein inhibitor kallistatin (red) and the proximal tubule marker *lotus tetragonolobus* lectin (LTL, green) shows distinct foci in the outer medulla of the kidney, where both kallistatin protein expression and the proximal tubule brush border integrity are lost in the P700 and LPS+P700-treated mice. Bottom: The renal cortex region of LPS+P700-treated mice displayed augmented kallistatin protein expression in dilated luminal compartments in the necrotic areas, where proximal tubule brush border integrity is lost. Images were acquired using a 20× objective. Boxed areas below each main image show individual staining (left, middle) and merged images (right). Nuclei were stained with Hoechst 33258 (blue). Scale bars: 100 µm. (B) Increased expression of BDKRB2 (red) was seen at tubular cell nuclear membranes in all treatment groups compared to controls. Cytoplasmic aggregation of BDKRB2 was seen in the necrotic areas of P700 and LPS+P700-treated groups. Proximal tubule brush borders were stained with LTL (green), nuclei with Hoechst 33258 (blue). Images were acquired using a 20× objective (top) or a 100× objective (bottom). Scale bars: 100 µm. (C) Transcription levels of *Kng2* (encoding kininogen 2 in mouse) and *F3* (encoding tissue factor) in kidney tissue measured by RT-qPCR. Red dots indicate the relative gene expression of each mouse compared to the control group. Black dots indicate the geometric mean per group, scaled as one in the control group. Benjamini-Hochberg-adjusted significance levels were calculated using the pairwise Mann–Whitney *U*-test (significance levels: one symbol *P*<0.05, two symbols *P*<0.01, three symbols *P*<0.001). Symbols used for significance: #, significance compared to the control group; $, significance compared to the LPS-treated group. ns: non-significant.

Since bradykinin receptor B2 (BDKRB2) and coagulation factor XII (F12, also known as FXII) have been reported to be requisite for PolyP-mediated pulmonary thrombus formation, we investigated related hemostatic mediators, such as components of the kallikrein–kinin system in kidney tissue (Müller et al., 2009; Zilberman-Rudenko et al., 2018). Immunostaining for the tissue kallikrein inhibitor kallistatin showed expression within the luminal compartments of the proximal tubules, partly overlapping the bush border marked by LTL in controls (Fig. 8A). While P100 caused no alterations, P700 caused a clear-cut loss of kallistatin protein expression within the proximal tubules, which also displayed a damaged brush border, in distinct foci within the outer medulla. LPS did not alter kallistatin expression compared to that in controls. LPS+P700-treated mice showed changes in the outer medulla similar to those seen in P700-treated mice. Additionally, the most severely affected cortical foci displayed increased kallistatin expression in the luminal compartments of dilated proximal tubuli, i.e. those no longer showing an intact proximal tubule brush border or typical overlap of kallistatin and LTL (Fig. 8A). BDKRB2 showed a diffuse tubular expression in the cytoplasm and near the nuclear membrane in the control group, whereas increased receptor expression was seen in all treatment groups in the nucleus and nuclear membrane. Cytoplasmic aggregates of BDKRB2 were seen in the most-damaged foci in the P700 and LPS+P700 groups (Fig. 8B). Transcription levels of kininogen-2 (*Kng2*) were elevated in the P100 and LPS+P100 groups, and elevated slightly in the P700 and LPS groups. Transcription levels of tissue factor (*F3*), the initiator of the extrinsic pathway of coagulation, was slightly increased in kidney tissue of all treatment groups but augmented in LPS+P700-treated mice in an amplificatory manner (Fig. 8C).

## DISCUSSION

Pleiotropic biological functions have been reported for the evolutionarily conserved PolyP polymers of various chain lengths both in eukaryotes and prokaryotes. As medium- or long-chains may be released to circulation, e.g. from platelets or bacteria, respectively (Schoeppe et al., 2024; Rashid et al., 2000; Ruiz et al., 2004; Krenzlin et al., 2023; Morrissey et al., 2012), we investigated the impact of intravenously administered medium-chain (P100) and long-chain (P700) PolyPs on kidney histopathology and cytokine levels, and assessed the synergistic effects of PolyPs and LPS on AKI development in BALB/c mice. We found that P700 induced glomerular microthrombi and slit diaphragm integrity loss, accompanied by albuminuria and tubular degeneration. Moreover, P700 augmented LPS-induced AKI as well as levels of cytokines in a synergistic manner. While LPS treatment alone induced diffusely distributed cortical histological lesions and upregulation of the inflammatory marker cJUN, with no signs of glomerular microthrombi, we showed that P700-mediated thrombotic damage is focal, likely affecting individual nephrons in the kidney.

In contrast to the pronounced adverse effects of P700 on the kidney, P100 showed only marginal effects in our analysis when administered alone or in combination with LPS. Accordingly, several studies have reported chain-length-dependent effects of PolyP exposure, with the longer chains having more capacity to induce coagulation, alter cytokine signaling, complement activity and mortality, but also to facilitate wound healing and gut epithelial cell function (Isozaki et al., 2021; Takauji et al., 2021; Pirttiniemi et al., 2023; Roewe et al., 2020, 2022; Terashima-Hasegawa et al., 2019; Smith et al., 2010). This is perhaps not surprising, as shorter chains at millimolar concentrations are endogenous in dense granules within mammalian platelets and can be secreted into plasma (Ruiz et al., 2004). PolyPs in the plasma form complexes with divalent cations (namely Ca^2+^) at the surface of cells, such as platelets, where they come into contact with plasma proteins (Huang et al., 2023; Morrissey et al., 2012). Of note, PolyPs are enzymatically cleaved by phosphatases and have a half-life of ∼2 h in the plasma *in vitro* (Morrissey et al., 2012; Lorenz et al., 1997)*,* although chain-length-dependent nanoparticle formation with cations may affect solubility and stability of PolyPs (Donovan et al., 2014). As monophosphate concentration in the blood is maintained at 2.5-4.5 mg/dl by excretion/reabsorption by the kidneys (Kestenbaum et al., 2005), it is plausible that PolyPs of shorter chain lengths are faster metabolized and cleared from the circulation, thereby diminishing their effects. Upon their release into circulation, long chain PolyPs may also bind to platelet surfaces and potentiate their thrombotic activity, as membrane-bound PolyPs can activate the contact pathway more efficiently than free soluble PolyPs (Verhoef et al., 2017). As the dose of PolyPs used in our study was based on previous *in vivo* studies of PolyP exposure (Roewe et al., 2020; Terashima-Hasegawa et al., 2019), more studies are needed to clarify serum PolyP concentration and chain lengths in healthy individuals as well as during microbial infections. Moreover, effective thresholds of PolyP dose and chain lengths require further assessment *in vivo*, considering the pleiotropic and highly context-dependent effects observed in exposure studies, which generally use few selected modal chain lengths of PolyPs, instead of a broader range of specifically determined chain lengths.

In this present study, intravenous P700 exposure induced focal glomerular microthrombi consisting of aggregated platelets, vWF, fibrin and collagen fibrils. The clot composition is characteristic of thrombotic microangiopathy that, in humans, can be caused by e.g. Shiga-toxin-producing *E. coli*, complement factor H-autoantibodies (hemolytic uremic syndrome, HUS), sepsis (disseminated intravascular coagulation, DIC) or deficiency of the vWF-cleaving metalloproteinase ADAMTS13 (thrombotic thrombocytopenic purpura, TTP) (Nuñez Zuno and Khaddour, 2024; Arnold et al., 2017). PolyPs participate in thrombosis/coagulation regulation through several mechanisms, such as activation of FXII/contact pathway of coagulation, and factor V, as well as binding and activation of vWF and interference of fibrinolysis (Huang et al., 2023; Zilberman-Rudenko et al., 2018; Smith et al., 2006, 2010; Montilla et al., 2012, 2022; Biswas et al., 2018). In the context of autoantibody-mediated thrombotic diseases, PolyPs have been shown to modify the conformation of platelet factor 4 (PF4) in manner similar to that of other polyanions, heparin (heparin-induced thrombocytopenia, HIT) and DNA (vaccine-induced immune thrombotic thrombocytopenia, VITT), which can, eventually, lead to platelet-activating autoantibodies (Huang et al., 2023; Brandt et al., 2014, 2015; Greinacher et al., 2021). Endogenous and microbial PolyPs of various lengths may, therefore, participate in thrombotic diseases through several routes, although autoantibody-mediated effects, such as thrombocytopenia, are not expected in our 20-h AKI model. Animal models of long-term PolyP exposure and recovery are still required to better define the role of PolyPs in the pathogenesis of acute and chronic forms of kidney injury.

The kallikrein–kinin system regulates hemostasis and inflammation in concert with the renin-angiotensin and complement systems (Bekassy et al., 2022; Pathak et al., 2013). We found increased nuclear expression of BDKRB2 in the kidney cortical tubular cells in all treatment groups. Knock-out mice for BDKRB2 or FXII have previously been reported to be protected from thrombosis and mortality induced by *E. coli*-derived PolyP (750 mg/kg, administered intraperitoneally) or platelet-derived PolyP (300 mg/kg, administered intravenously) (Müller et al., 2009). On the other hand, simultaneous knock-out of the constitutively expressed BDKRB2 and the inducible bradykinin receptor B1 (BDKRB1) have been shown to exacerbate AKI in an ischemia reperfusion injury mouse model (Kakoki et al., 2007), as well as diabetic nephropathy development in Akita mice (Kakoki et al., 2010). These studies suggest that BDKRB2 and BDKRB1 have protective roles by limiting oxidative stress, mitochondrial DNA damage and expression of fibrogenic genes in the kidney (Kakoki et al., 2007, 2010). We showed here that renal expression of the tissue kallikrein inhibitor kallistatin is lost in the disrupted proximal tubule brush border and increased in the dilated lumen within the most severely affected foci in mice treated with P700 alone or with LPS+P700. Accordingly, kallistatin has been shown to be protective in chronic kidney disease and sepsis-induced tissue injury, by inhibiting oxidative stress, fibrosis and inflammation (Chao et al., 2016; Yiu et al., 2016, 2021).

Moreover, we found focally affected glomeruli with granular deposits of two slit diaphragm components – i.e. of the transmembrane junction protein nephrin that is expressed in podocytes, and of the tight junction protein ZO1 – in P700-treated as well as LPS+P700-treated mice. The degree of nephrin granularity correlated with the glomerular vWF-positive area in the PolyP-treated groups, indicating a connection between glomerular microthrombi and granular phenotype of the slit diaphragm components. However, some clots may lack vWF or may have been resolved in our model, as nephrin granularity was seen also without vWF-positive clots in the P700 and LPS+P700 groups. Analysis of ultrastructural changes of glomeruli revealed large electron-dense vesicles characteristic of lysosomes in the podocyte cytoplasm in response to treatment with P700 or LPS+P700. The slit diaphragm forms a dynamic zipper-like structure, which participates in the highly selective filtration/retention processes, but the intracellular domain of nephrin is also connected to cellular signaling, mediating podocyte mechanotransduction, cytoskeleton motility and endosomal vesicle trafficking (Martin and Jones, 2018; Grahammer et al., 2013, 2016; Li et al., 2020; Meyer-Schwesinger, 2021). Interestingly, in Zucker diabetic fatty rats a granular nephrin phenotype has been reported, possibly due to increased expression of its interaction partner PACSIN2, which enhances nephrin trafficking *in vitro* (Dumont et al., 2017). Our study indicates that the P700-mediated podocyte injury and nephrin granularity may be connected to cellular stress mechanisms, such as endocytic recycling and lysosomal degradation following glomerular congestion and thrombus formation. However, further studies are needed to show whether the highly negatively charged P700 has direct effects on the slit diaphragm filtration or lysosome swelling in podocytes.

The P700-treated mice showed varying degrees of tubular damage in conjunction with the foci of affected glomeruli. Notable histological lesions, such as acute tubular necrosis, proximal tubule brush border disruption and apoptosis in the outer medulla were observed in the most severely affected P700-treated mice. However, some albuminuria, nephrin granularity and cJUN-positive tubular foci were observed in all P700-treated mice, including those displaying no clear signs of tubular degeneration. This further supports our hypothesis that the glomerulus is a primary site of injury by P700 due to thrombus formation, leading to glomerular congestion, podocyte injury and albuminuria. Tubular damage is likely secondary, since proximal tubules and the medulla are often early sites of damage in AKI, as the highly metabolically active tubular epithelial cells are sensitive to hypoxia, and disturbances in tubular fluid flow and shear stress (Birdsall and Hammond, 2021; Basile et al., 2012). However, the potential direct effects of P700 on tubular cell function still need to be elucidated. Given the variability observed in the PolyP-mediated pathological manifestations in our study, thorough assessment of the kidney injury mechanisms will benefit from larger studies performed with more animals.

While P700 affected only isolated foci in the kidney cortex and outer medulla, LPS caused more diffusely distributed histological lesions and expression of cJUN in the cortex. In the P700-treated mice the glomerular nephrin granularity correlated with vWF-positive area and coincided with tubular cJUN expression; however, LPS-treated mice did not show similar correlation or any signs of thrombosis in general, indicating that LPS induces the observed milder degrees of nephrin granularity through a different mechanism. Assessment of the synergistic effects of PolyPs and LPS showed that histologically detectable acute tubular necrosis, albuminuria, *Lcn2* transcription and cytokine levels were exacerbated in response to LPS+P700 treatment compared to treatment with LPS or P700 alone. Immunothrombosis is a concept describing the connection between coagulation and the innate immune system, which operates though the tissue factor -mediated extrinsic pathway of coagulation in an amplificatory manner (Marcos-Jubilar et al., 2023). In our study, P700 treatment alone increased transcription levels of tissue factor-encoding *F3* to levels similar to those after treatment with LPS only but did not cause cytokine release or clinical signs of sepsis as was seen with LPS treatment. This highlights the crucial role of initial activation of pattern recognition receptors within the innate immune system before the tissue factor-mediated amplificatory immunothrombosis can occur (Marcos-Jubilar et al., 2023). While tissue factor is best known for amplifying the coagulation process under pathological conditions (Pawlinski et al., 2004; Grover and Mackman, 2018; Marcos-Jubilar et al., 2023), the function of tissue factor is bidirectional, as it can also amplify cytokine signaling, e.g. through protease-activated receptors (PARs) (Heuberger and Schuepbach, 2019), inflammasome activation and neutrophil extracellular traps (NETs) (Yang et al., 2020). PolyPs have also been connected to the induction of NETs (Chrysanthopoulou et al., 2017) that, as negatively charged chromatin structures, can further activate the contact pathway and function as a scaffold for platelets, red blood cells, extracellular vesicles, vWF and tissue factor (Marcos-Jubilar et al., 2023; Yang et al., 2020). Comprehensive understanding of various PolyP-associated hemostatic amplificatory mediators still requires extensive investigation *in vivo*.

Our study highlights the importance of thorough mechanistic characterization of individual players behind kidney damage, considering the diversity of kidney injury manifestations seen in animal models and of kidney injury types seen in humans. However, since we were limited by the small volume of urine and blood samples, we were unable to extensively analyze, e.g. plasma coagulation factors, changes in leukocyte and platelet function or urine and serum markers, in the mice. Urine and serum protein markers, such as LCN2 (early-stage marker for glomerular and tubular injury) and KIM-1 (marker for proximal tubule injury), have been proposed to better describe the severity, prognosis and specific site of injury within the kidney in humans (Turgut et al., 2023; Han et al., 2002; Brilland et al., 2023; Panduru et al., 2015; Limonte et al., 2020). Interestingly, in mouse kidney tissue we found *Lcn2* transcription to be notably more sensitive to LPS than to P700, while *Kim-1* transcription responded to LPS and P700 similarly. On the other hand, *Kim-1* transcription was decreased in the LPS+P700 group, which may be a result of the marked proximal tubule injury and apoptosis in the group. *Kim-1* acts as a scavenger in the phagocytosis of apoptotic debris in the proximal tubules (Zhang and Liu, 2025) and, accordingly, transcription of *Hmox1* was augmented in the LPS+P700 group, indicating increased oxidative stress.

Sufficient treatment options for sepsis-related mortality and organ damage are still lacking, and anticoagulative tissue factor or FVII inhibitors have been unsuccessful in human trials, despite promising preclinical trials in animals (Grover and Mackman, 2018; Creasey et al., 1993; Abraham et al., 2003; Welty-Wolf et al., 2006; Vincent et al., 2009). Although administration of alkaline phosphatases, which dephosphorylate/detoxify various compounds, such as LPS and PolyP, has shown marginal capacity to ameliorate sepsis-induced AKI in humans (Tang et al., 2020; Pickkers et al., 2024; Baek et al., 2018), further studies are needed to map out treatment options that better consider the timing and type of AKI. Moreover, there is need for treatments to improve kidney function after injury has already occurred and to prevent progression into chronic kidney disease, which may include better management of blood coagulation and inflammation (Iba et al., 2024). In conclusion, P700 treatment may be used as an effective experimental model to study systemic effects of renal thrombosis, characteristic of thrombotic microangiopathy (commonly modelled by depletion of ADMTS13 or administration of bacterial toxins, e.g. Shiga toxin and LPS, in animals) (Arnold et al., 2017; Richards and Kavanagh, 2009), while, in combination with LPS, the pro-inflammatory phenotype is augmented in a manner characteristic of immunothrombosis (Marcos-Jubilar et al., 2023). PolyPs serve also as interesting treatment targets in humans; although, how much PolyPs contribute to thrombosis in the context of bacterial infections or tissue damage in humans remains to be elucidated.

## MATERIALS AND METHODS

### Experimental animals and procedures

The study was conducted adhering to the ethical guidelines, and the ethical permit has been approved by the Finnish Project Authorisation Board (ELLA, ESAVI/6504/2020). The study was conducted using 9-week-old wild-type male BALB/c mice (BALB/cAnNCrl; Scanbur, Karlsunde, Denmark) housed in pairs in individually ventilated cages with environmental enrichment, 12:12 h light-dark cycle, and free access to water and chow. Acute kidney injury (AKI) was induced by 5 mg/kg intraperitoneal injection of LPS (from *E. coli* strain O111:B4, Sigma Aldrich, Germany). Based on previous PolyP-exposure studies (Roewe et al., 2020; Terashima-Hasegawa et al., 2019) a dose of 10.2 mg/kg (equivalent of 100 µmol/kg of total phosphate residues) of medium-chain (P100) PolyPs (heterogenous size distribution of 40-160 phosphate units; Kerafast, Newark, CA, USA) or long-chain (P700) PolyPs, (heterogenous size distribution of 99–1298 phosphate units; Kerafast) were administered by intravenous injection under isoflurane anesthesia. As requested by ELLA, the used LPS and PolyP concentrations were tested on a smaller number of mice (*n=*2-3 per group) in a pilot experiment (24-h follow-up for LPS treatment and 3-day follow-up for P100 and P700 treatment). Based on the albuminuria levels in the pilot experiment (data not shown), the number of mice per group in the final experiment was kept to a minimum, with which reliable results were attainable.

The treatment groups in the final experiment were untreated control (*n=*8), P100-treated (*n=*4), P700-treated (*n*=4), LPS-treated (*n*=8), LPS+P100-treated (*n*=8) and LPS+P700-treated (*n*=8) mice. The mice were sacrificed 20 h after injection, and the kidneys, sera and urine were collected for subsequent analysis. Two mice in the LPS+P700 treatment group in the final experiment unexpectedly died prior to sample collection and these mice were not included in subsequent analysis of urine or serum samples, but kidney tissue samples were included in further analysis.

### Urine albumin and creatinine measurement

Spot urine was collected prior to sacrificing the mice; however, urine was not attainable for three mice in the LPS+P700 treatment group and one mouse in the LPS treatment group due to anuria. Urinary albumin concentrations were measured with ELISA (Bethyl Laboratories, Montgomery, TX, USA; cat no.: E99-134) and creatinine was measured using a Creatinine Urinary Detection Kit (Thermo Fisher Scientific; cat no.: EIACUN).

### Serum sample preparation and Quansys multiplex cytokine assay

Blood samples were collected during terminal anesthesia by cardiac puncture. 0.5-1 ml blood was pipetted into serum separator tubes and the tube inverted to dissolve the clotting activator. The tubes were incubated for 30 min at room temperature before centrifuging at 10,000 ***g*** for 5 min. Serum cytokine levels were assayed with the Q-Plex Mouse Cytokine Panel 1, 6-Plex (Quansys Biosciences, Logan, UT, USA). Serum samples were diluted 1:2 into the kit sample diluent and the plate was measured with a chemiluminescent reader (Q-view imager LC, Quansys Biosciences).

### Kidney tissue sample preparation for histological staining

The dissected kidneys were cut in sagittal and horizontal plane into four pieces for subsequent processing. For histological analysis, the tissues were fixed with 10% neutral-buffered formalin for 24 h, after which they were stored in 70% ethanol at +4°C from 24 h to 72 h before tissue processing, paraffin embedding, and sectioning at the Finnish Centre for Laboratory Animal Pathology (FCLAP).

### Histochemical staining and digital slide scanning of kidney sections

Hematoxylin & eosin (H&E), Periodic acid–Schiff (PAS), Masson's trichrome stain and von Kossa staining for the paraffin sections from all 40 mice were performed by the Finnish Centre for Laboratory Animal Pathology (FCLAP). Whole-slide scan images (40× air objective) of H&E, PAS and Masson's trichrome stained microscopy slides were generated using a 3DHISTECH Pannoramic 250 FLASH II digital slide scanner at the Genome Biology Unit, supported by Helsinki Institute of Life Science, Helsingin Yliopisto (HiLIFE) and the Faculty of Medicine, University of Helsinki, and Biocenter Finland; cropped images were acquired and adjusted with SlideViewer 2.6.0 (3DHISTECH).

### Immunohistochemical staining of kidney sections and quantification

Paraffin-embedded 4 µm tissue sections were deparaffinized/rehydrated with a standard xylene-ethanol series. Antigen retrieval was conducted with 20 min gentle boiling in 10 mM Tris, 1 mM EDTA pH 9+0.05% Tween in phosphate-buffered saline (PBS). Once cooled the slides were rinsed with PBS and permeabilized/blocked for 1 h at room temperature in 5% fetal bovine serum (FBS, Thermo Fisher Scientific), 0.1% Triton X-100 (Sigma Aldrich) in PBS. Primary antibodies (Table S3) were incubated overnight at +4°C in 1% FBS in PBS, after which they were washed three times for 15 min in the same buffer. Secondary antibodies (anti-rabbit IgG Alexa Fluor 594; Thermo Fisher Scientific, cat. no.: A-21207 and anti-Guinea Pig IgG Alexa Fluor 488, cat. no.: A-11073; both diluted 1:500) or fluorescein-conjugated *lotus tetragonolobus* lectin (LTL; Vector Laboratories; see Table S3) were incubated for 1 h at room temperature before the slides were washed three times for 15 min. Staining of nuclei with Hoechst 33258 (Thermo Fisher; cat. no.: H1398) was included during the secondary antibody incubation. Autofluorescence quenching was conducted with TrueView (Vector Laboratories, cat. no.: SP-8400) before the slides were mounted with VectaShield Vibrance mounting medium (Vector Laboratories, cat. no.: H-1700). Images were acquired with Axio Imager M2 microscope, equipped with Axiocam 503 (Carl Zeiss Microscopy GmbH, Oberkochen, Germany) using Zen 3.1 Imaging software.

For nephrin- and ZO1-stained glomeruli the degree of granularity was assessed by observers unaware of the experimental conditions, on a scale from 0-5 for granularity (4-8 mice per group, 7-27 imaged glomeruli per mouse, 444 imaged glomeruli in total; images were acquired using a 100× objective). Kidney sections were stained for the inductor of platelet adhesion von Willebrand factor (vWF) (3-4 mice per group, 6-11 imaged glomeruli per mouse, 147 imaged glomeruli in total; images were acquired using a 100× objective) and the macrophage marker protein F4/80 (4-8 mice per group, 3-9 imaged cortical frames per mouse, images were acquired using a 20× objective), cell covered area per frame was quantified with Fiji ImageJ (Schindelin et al., 2012). For all other immunohistochemistry markers assessing qualitative changes, at least three frames were included (acquired by using a 20× objective from 3-4 mice per group).

### Transmission electron microscopy sample preparation and analysis

For transmission electron microscopy (TEM) analysis of glomeruli, 1-2-mm cubes from the kidney cortex were fixed in 2.5% glutaraldehyde, 2.5% paraformaldehyde, 0.1 M phosphate buffer pH 7.4 for 3 h. The samples were further processed by the Electron Microscopy Unit at HiLIFE, Institute of Biotechnology (University of Helsinki and Biocenter Finland), as described previously (Wang et al., 2024) and imaged using JEM-1400PLUS (JEOL Ltd, Japan) with an Orius SC 1000B bottom-mounted charge-coupled device camera (Gatan, Inc., Pleasanton, CA, USA). Glomeruli from treatment groups, i.e. controls, P700, LPS and LPS+P700, were imaged and assessed at 1200× and 3000× magnification. The diameter of electron-dense vesicles characteristic of lysosomes, multivesicular bodies or peroxisomes (Neikirk et al., 2023) was quantified from 1200× magnification images using Fiji ImageJ (Schindelin et al., 2012) (three mice per group, between three and seven imaged glomeruli per mouse, one to five frames per glomerulus).

### RT-qPCR

Kidney tissue was lysed in a bead homogenizer (Analytik Jena, Jena, Germany) and RNA was extracted using the NucleoSpin RNA kit (Macherey-Nagel, Düren, Germany). 1 µg of RNA was reverse transcribed into cDNA with iScript cDNA Synthesis Kit (Bio-Rad) and further diluted 1:10 in molecular biology grade water. For reverse transcription–quantitative polymerase chain reaction (RT-qPCR) 2 µl of template and 0.25 µM of forward and reverse primers together with iTaq Universal SYBR Green Supermix (Bio-Rad) were used in a total PCR reaction volume of 10 µl. Hard-Shell 384-Well PCR Plates (Bio-Rad) and the CFX384 Touch Real-Time PCR Detection System thermal cycler (Bio-Rad) were used with the following program: initial denaturation at 95°C for 30 s, followed by 40 cycles of denaturation at 95°C for 5 s, and annealing at 60°C for 30 s. Fold-changes in gene expression were analyzed based on previously described methods (Hellemans et al., 2007; Vandesompele et al., 2002) and normalized to housekeeping genes *Eef2* and *S18*. Primer sequences with original references are presented in Table S4. New primers were generated using Primer3web version 4.1.0 and Primer-BLAST.

### Statistical analysis

R version 4.2.3 was used for statistical analysis. Pairwise Mann–Whitney *U*-test with Benjamini–Hochberg adjusted *P*-values was used for two-group comparisons and Spearman's rank correlation coefficient was used to assess the correlation between glomerular markers.

## Supplementary Material

10.1242/dmm.052361_sup1 Supplementary information

Table S5. Mouse urine albumin and creatinine concentrations

Table S6. Mouse serum cytokine concentrations

Table S7. Kidney tissue RT-qPCR Ct values

Table S8. Kidney cortex F4/80-positive area per frame

Table S9. Glomerular Nephrin and ZO1 granularity scores

Table S10. Glomerular vWF-positive area and Nephrin granularity scores

Table S11. Glomerular TEM image frames with quantified vesicle diameter
