# Supplementary material for: Long-chain polyphosphates induce glomerular microthrombi and exacerbate LPS-induced acute kidney injury in mouse
Source: Dis Model Mech. 2026 Mar 30;19(3):dmm052361. doi: 10.1242/dmm.052361 (PMC13054936; doi:10.1242/dmm.052361)
Supplement: Supplementary information [file dmm-19-052361-s1.pdf]

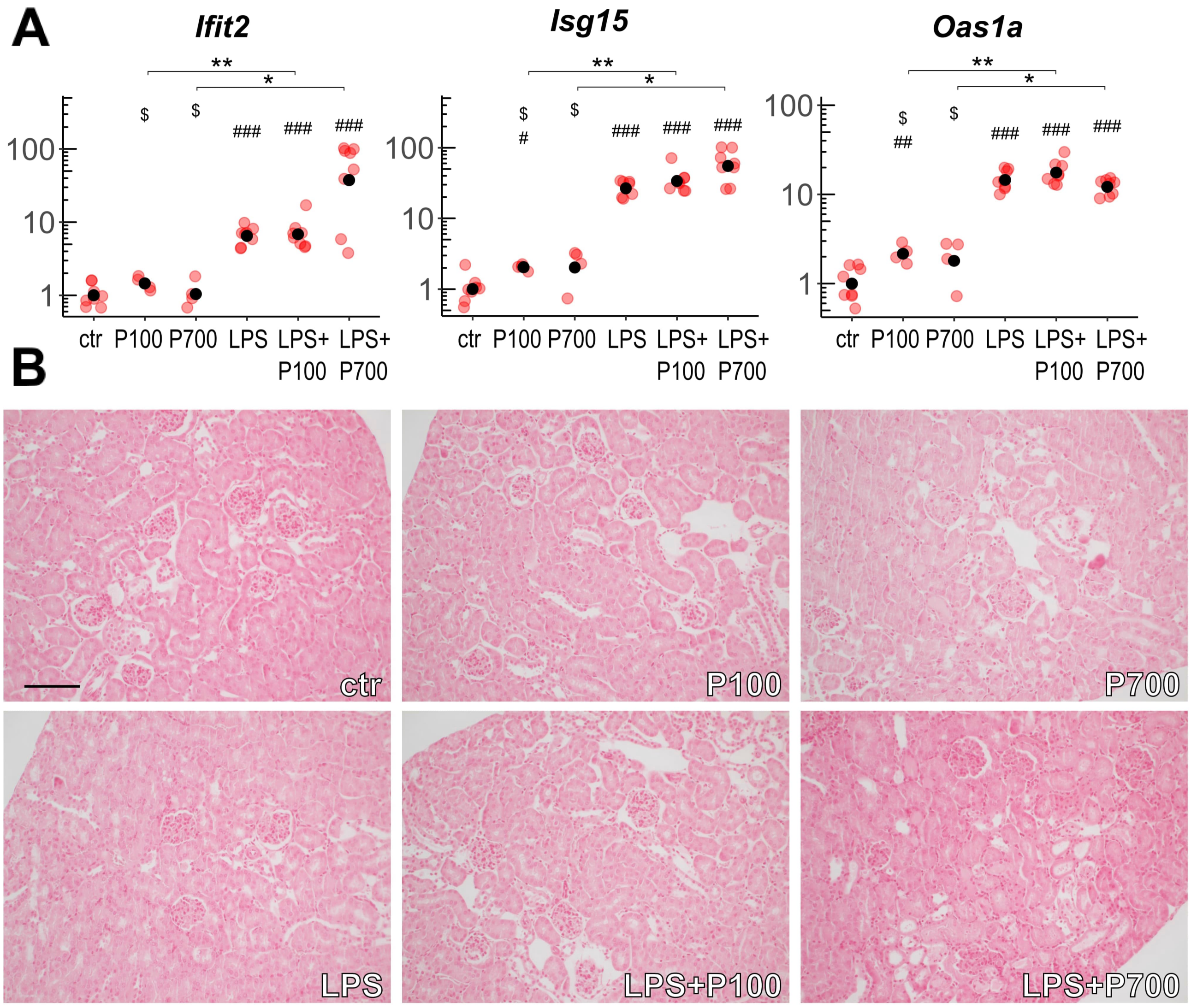

Fig. S1. Kidney transcription of interferon stimulated genes and Von Kossa staining of kidney cortex of polyphosphate and LPS-treated mice. (a) Transcription of interferon stimulated genes, *Ifit2*, *Isg15*, and *Oas1a*, measured by RT-qPCR from the kidney tissue of mice were treated with medium-chain polyphosphates (P100), long-chain polyphosphates (P700) alone and in combination with LPS. Red dots indicate the relative gene expression of each mouse compared to control group. Black dots indicate the geometric mean per group, scaled as one in the control group. Benjamini-Hochberg adjusted significance levels are calculated using the Mann-Whitney U test., *: $p < 0.05$, **: $p < 0.01$, ***: $p < 0.001$. Symbols used for significance: #: significance compared to the control group, $: significance compared to the LPS-treated group. (b) The Von Kossa staining showed no calcium deposits, characteristic of phosphate toxicity in the kidney tissue of mice treated with P100 or P700 alone or in combination with LPS. Scale bar 100µm.

Disease Models & Mechanisms • Supplementary information

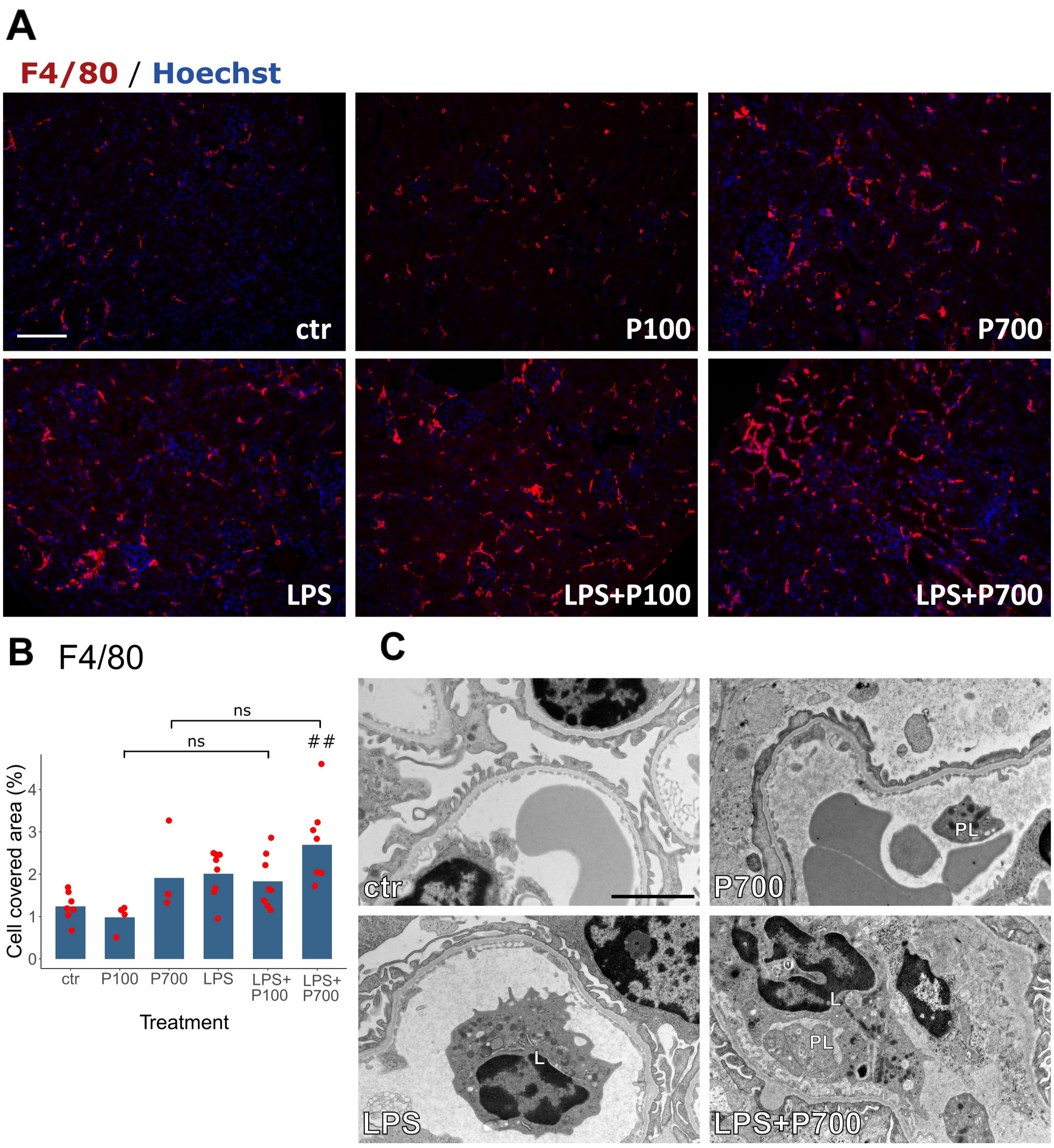

Fig. S2. Leukocyte infiltration in the kidney cortex and glomerular capillaries of mice treated with long-chain polyphosphates and LPS. (a) Representative images of tissue resident macrophage infiltration in the kidney cortex of mice treated with medium-chain polyphosphates (P100), long-chain polyphosphates (P700) alone and in combination with LPS, immunostained with F4/80 (red). Nuclei stained with Hoechst (blue), scale bar 100 µm. (b) Macrophage covered area in the kidney cortex. Red dots represent the mean leukocyte covered area in a frame per mouse and the bars represent the mean values per group. Benjamini-Hochberg adjusted significance levels are calculated using the pairwise Mann-Whitney U test., **: $p < 0.01$. Symbols used for significance: #: significance compared to the control group (n: 4-8 mice per group, 3-9 frames per mouse). (c) Transmission electron microscopy imaging shows intracapillary leukocytes in mice treated with LPS alone and in combination with long-chain polyphosphates (P700). 3000x magnification, scale bar 2 µm, platelets (PL), leukocytes (L).

Disease Models & Mechanisms • Supplementary information

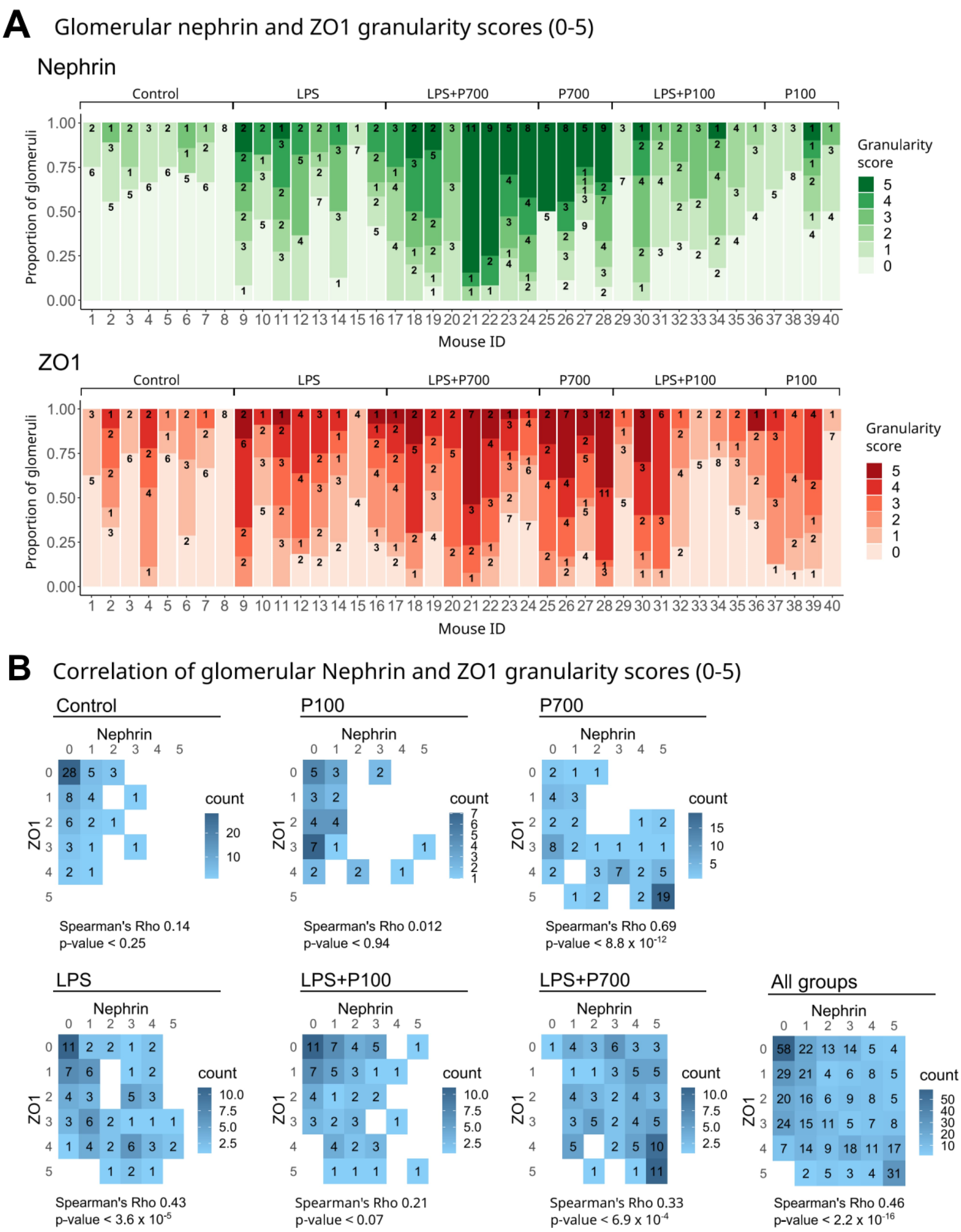

Fig. S3. Glomerular nephrin and ZO1 granularity score proportions and correlation in polyphosphate and LPS-treated mice. (a) Proportions of nephrin and ZO1 granularity for each mouse treated with medium-chain polyphosphates (P100), long-chain polyphosphates (P700) alone and in combination with LPS. Stacked bars represent the proportion of glomeruli affected with granularity (scored 0-5 from immunostained images) with the number of assessed glomeruli indicated inside the bars. (b) Heatmap contingency tables and spearman's correlation coefficients of glomerular nephrin and ZO1 granuarity scores (n: 4-8 mice per group, 7-27 imaged glomeruli per mouse, 444 glomeruli in total).

Disease Models & Mechanisms • Supplementary information

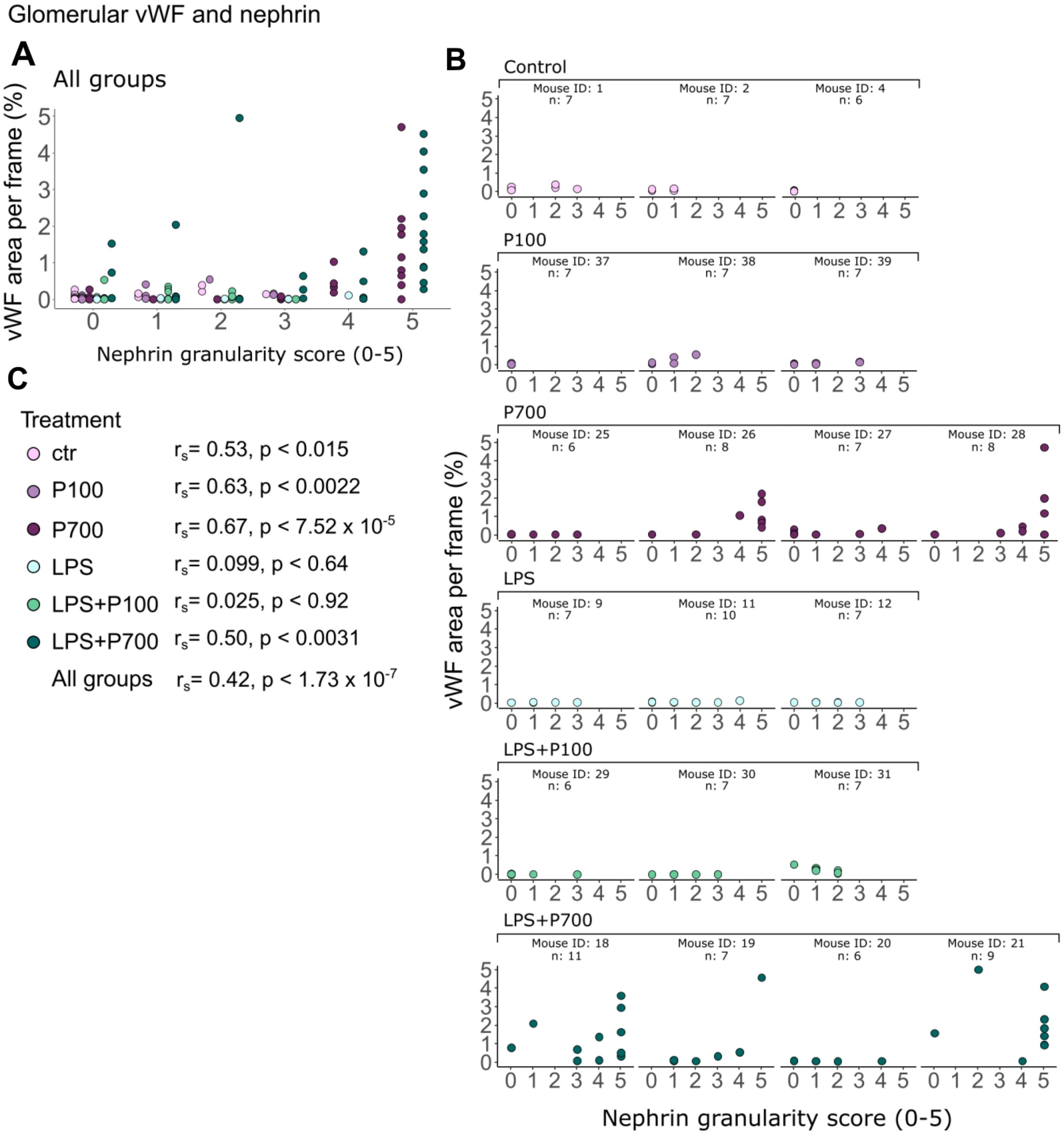

Fig. S4. Glomerular von Willebrand factor area and nephrin granularity scores in polyphosphate and LPS-treated mice. Quantification of von Willebrand factor-positive (vWF) area per frame and nephrin granularity score (scored 0-5 from immunostained images) for mice treated with medium-chain polyphosphates (P100), long-chain polyphosphates (P700) alone and in combination with LPS. Each dot represents one imaged glomerulus (n: 3-4 mice per group, 6-11 imaged glomeruli per mouse), (a) all groups combined, (b) number of assessed glomeruli indicated for each mouse, (c) correlation of glomerular vWF-positive area and nephrin granularity score (Spearman's correlation coefficient, r_s).

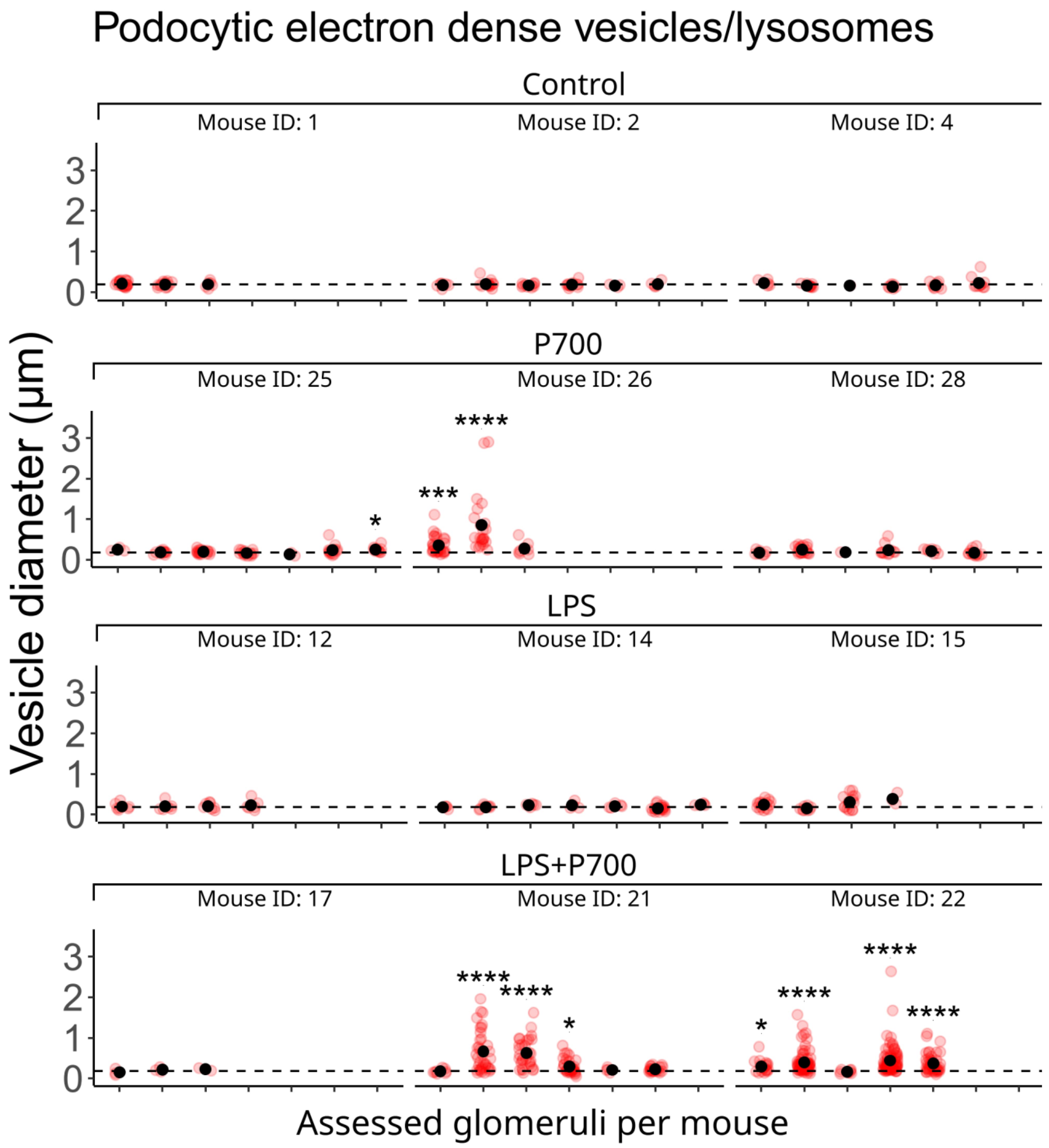

Fig. S5. Diameter of podocytic vesicles characteristic of lysosomes, multivesicular bodies or other endosomes in transmission electron microscopy analysis. Quantification of the diameter of podocytic vesicles characteristic of lysosomes/multivesicular bodies/endosomes in mice treated with long-chain polyphosphates (P700) and LPS. Red dots indicate individual vesicles measured in glomeruli. Black dots indicate the mean vesicle diameter per glomerulus. Benjamini-Hochberg adjusted significance levels are calculated for each glomerulus compared to the mean glomerular vesicle diameter of the healthy controls (0.18 µm, dotted line) using the Mann-Whitney U test., *: $p < 0.01$, ***: $p < 0.001$, ****: $p < 0.0001$ (n: 3 mice per group, 3-7 imaged glomeruli per mouse, 1-5 frames per glomerulus).

Disease Models & Mechanisms • Supplementary information

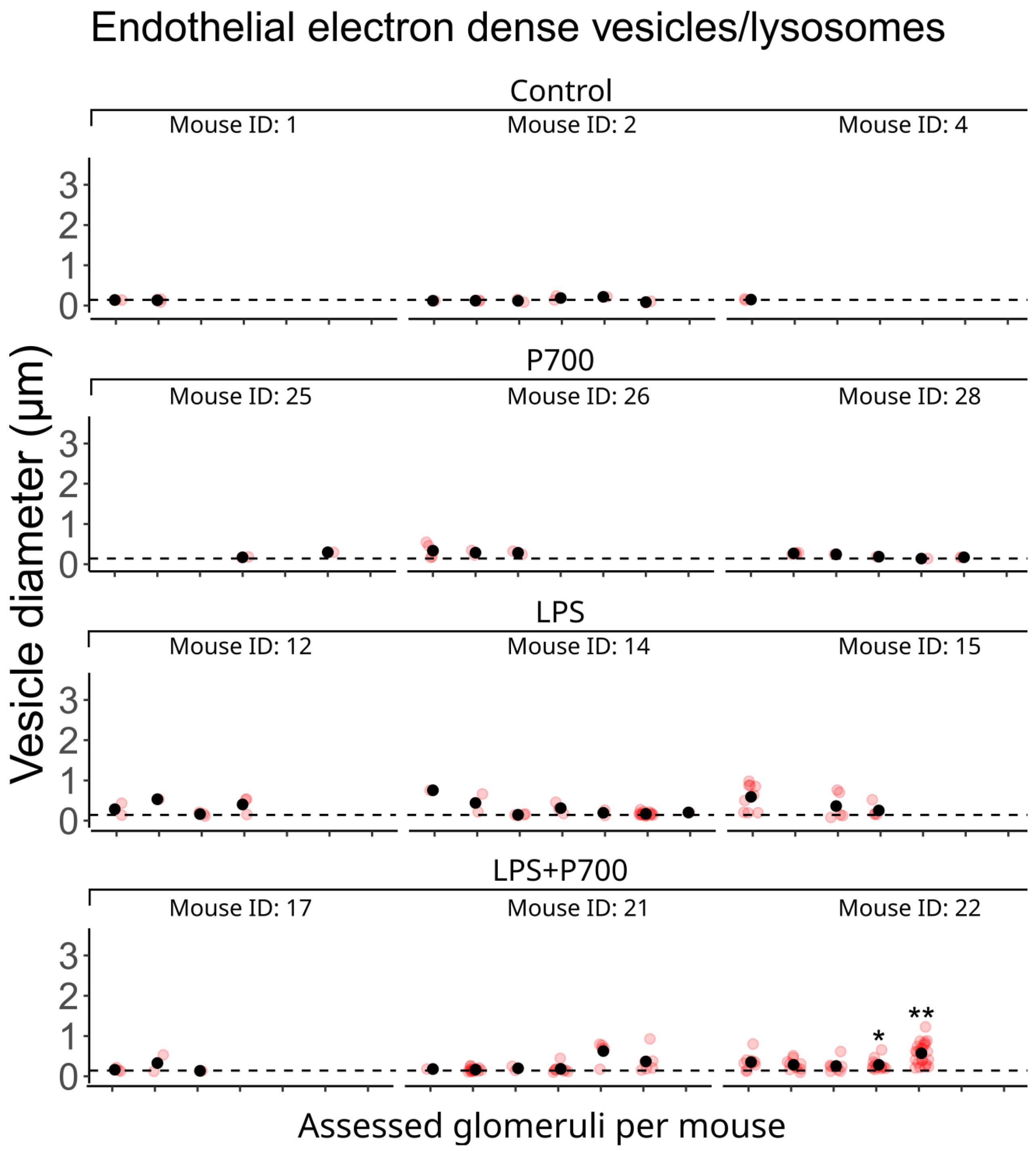

Fig. S6. Diameter of glomerular endothelial cell vesicles characteristic of lysosomes, multivesicular bodies or other endosomes in transmission electron microscopy analysis. Quantification of the diameter of endothelial cell vesicles characteristic of lysosomes/multivesicular bodies/endosomes in mice treated with long-chain polyphosphates (P700) and LPS. Red dots indicate individual vesicles measured in glomeruli. Black dots indicate the mean vesicle diameter per glomerulus. Benjamini-Hochberg adjusted significance levels are calculated for each glomerulus compared to the mean glomerular vesicle diameter of the healthy controls (0.14 µm, dotted line) using the Mann-Whitney U test., *: $p < 0.05$, **: $p < 0.01$ (n: 3 mice per group, 3-7 imaged glomeruli per mouse, 1-5 frames per glomerulus).

Disease Models & Mechanisms • Supplementary information

Table S1. Histopathological changes seen in H&E, PAS, and Masson's Trichrome stained sections.

Treatment group	Mouse ID	Tubular necrosis/ degeneration	Glomerular thrombus formation	Pathological changes
ctr	1	0	0	
ctr	2	0	0	
ctr	3	0	0	
ctr	4	0	0	
ctr	5	0	0	
ctr	6	0	0	
ctr	7	0	0	
ctr	8	0	0	
LPS	9	1	0	See mouse 13.
LPS	10	1	0	See mouse 13.
LPS	11	1	0	See mouse 13.
LPS	12	1	0	See mouse 13.
LPS	13	1	0	Minimal tubular degeneration in multiple foci in the subcapsular cortex. The degenerated tubule cells are mildly swollen and show increased eosinophilia and foamy or vacuolated cytoplasm.
LPS	14	1	0	See mouse 13.
LPS	15	1	0	See mouse 13.
LPS	16	1	0	See mouse 13.
LPS+P700	17	1	0	See mouse 13. + A focal acute coagulative necrosis with some hemorrhages and neutrophil infiltration. Oblong focal necrosis extends from OSOM to cortex. Not present in PAS- or MT-stained slides.
LPS+P700	18	3	1	See mouse 21. Glomerular capillary congestion impedes detection of the thrombi.
LPS+P700	19	1	0	See mouse 13.
LPS+P700	20	1	0	Minimal tubular degeneration in multiple small subcapsular foci. The degenerated tubular epithelial cells show foamy, granular or vacuolated cytoplasm (See 13). Necrotic, exfoliated cells in one tubular segment in the OSOM in one HE section - focal change.
LPS+P700	21	3	2	Moderate / marked acute tubular necrosis. Multifocal cortical, subcapsular and single OSOM segments of tubules showing granular brightly eosinophilic cytoplasm, karyorhexis or -lysis and

Disease Models & Mechanisms • Supplementary information

				cell sloughing. Tubular protein casts. Eosinophilic casts / globules (fibrin thrombi) and erythrocyte aggregates in the glomerular capillary loops in several glomeruli.
LPS+P700	22	4	2	Marked tubular necrosis. Multifocaltubular necrosis extends to ISOM and inner medulla.
LPS+P700	23	3	1	See mouse 21. Glomerular congestion impedes cast assessment. Marked glomerular congestion.
LPS+P700	24	3	1	See mouse 21. Little medulla present in the section (only some OSOM). Marked glomerular congestion.
P700	25	0	0	
P700	26	2	2	Mild tubular degeneration and necrosis. Some cortical, subcapsular and OSOM tubular segments showing granular brightly eosinophilic cytoplasm, karyorhexis or -lysis and cell sloughing or degenerative changes. Protein casts m the collecting ducts or tubules (pars recta) and the collecting ducts m inner medulla. Glomerular congestion. Eosinophilic casts / globules (fibrin thrombi) and erythrocyte aggregates m the glomerular capillary loops in several glomeruli.
P700	27	1	1	Single deep cortical or OSOM degenerated tubular segments (few necrotic cells). See mouse 13.
P700	28	2	1	See mouse 26. Eosinophilic casts / globules (fibrin thrombi) and erythrocyte aggregates m the glomerular capillary loops in some glomeruli.
LPS+PlOO	29	1	0	See mouse 13.
LPS+PlOO	30	0	0	
LPS+PlOO	31	0	0	
LPS+PlOO	32	0	0	Focal tubular degeneration in the OSOM in HE. Not present in PAS- or MT-stained slides.
LPS+PlOO	33	1	0	See mouse 13. Dilated glomerular capillaries.
LPS+PlOO	34	1	0	See mouse 13.
LPS+PlOO	35	1	0	See mouse 13.
LPS+PlOO	36	0	0	
PlOO	37	0	0	
PlOO	38	0	0	
PlOO	39	0	0	
PlOO	40	0	0	

Note: Mice 23 and 24 were found dead before sample collection. Hematoxylin & Eosin staining (HE), Periodic Acid-Schiff (PAS), Masson's Trichrome staining (MT), outer stripe of the outer medulla (OSOM), inner stripe of the outer medulla (ISOM). Grading of tubular necrosis/degeneration and glomerular thrombus formation; 0: no change, 1: minimal, 2: mild, 3: moderate, 4: marked.

Table S2. Number of affected glomeruli with observed intracapillary clots, leukocytes, and podocyte vacuolization in transmission electron microscopy analysis.

Treatment group	Mouse ID	Adhered platelets/ platelet clots	Fibrin strands/ collagen fibrils	Intracapillary leukocytes	Podocyte vacuolization	Assessed glomeruli in total per mouse
ctr	1	0	0	0	0	3
	2	0	0	0	0	6
	4	0	0	0	0	6
P700	25	0	0	0	0	7
	26	2	0	0	2	3
	28	2	0	0	0	6
LPS	12	0	0	1	0	4
	14	0	0	4	0	7
	15	0	0	1	2	4
LPS+P700	17	0	0	0	0	3
	21	3	0	0	0	6
	22	4	3	1	1	5

Table S3. Primary antibodies used in immunohistochemical staining. Target Host

Target	Host Animal	Manufacturer	Catalogue number	Used dilution
Proximal tubule - Lotus Tetragonolobus Lectin (LTL)	-	Vector Laboratories	#FL-1321	1:200
Cleaved caspase-3	Rabbit	Cell Signaling Technology	#9661	1:200
Integrin p3 (CD61)	Rabbit	Cell Signaling Technology	#13166	1:250
Von Willebrand factor (vWF)	Rabbit	Novus Biologicals	#NB600-586	1:1000
Nephrin	Guinea pig	Progen	#GP-N2	1:200
Zona Occludens 1 (ZOl)	Rabbit	Abeam	#ab221546	1:1000
Bradykinin Receptor B2 (BDKRB2)	Rabbit	Novus Biologicals	#NBPl-46328	1:200
Kallistatin	Rabbit	ThermoFisher	#PA5-96636	1:100
cJUN	Rabbit	Cell Signaling Technology	#9165	1:200
Interferon regulatory factor 1 (IRF1)	Rabbit	Cell Signaling Technology	#8478	1:100
CD45	Rabbit	Cell Signaling Technology	#70257	1:150
F4/80	Rabbit	Cell Signaling Technology	#70076	1:150

Disease Models & Mechanisms • Supplementary information

Table S4. Primer sequences used in RT-qPCR.

Gene name	Forward primer	Reverse primer	Reference
Kim-1	5'-TTGCCTTCCGTGTCTCTAAG-3'	5'-AGATGTTGTCTTCAGCTCGG-3'	S1
Lcn2	5'- CCACCACGGACTACAACCAG -3'	5'-AGCTCCTTGGTTCTTCCA TACAG -3'	-
Hmoxl	5'-CACATCCAAGCCGAGAATGC-3'	5'-AAGGAAGCCATCACCAGCTTAAA-3'	-
Il1b	5'- CTCCAGCCAAGCTTCC TTGT-3'	5'- TCATCACTGTCAAAAGGTGGCA -3'	S2
Tnfa	5'-TGGCACCACTAGTTGGTTGTCT -3'	5'-AGCCTGTAGCCCACGTCGTA -3'	S1
Il6	5'-ATCGTGGAAATGAGAAAAGAGTTGT-3'	5'- CTGCAAGTGCATCATCGTTGT -3'	-
Nphsl	5'- AGAAGCTCCACGGTTAGCAC -3'	5'- CCTGTGAAGGCTTGGCGA TA -3'	-
Podxl	5'- TGCAACAGTCTATGGCGTCT -3'	5'- GGAGGTTCACAGTTTAGCTGGT -3'	-
F3	5'-CAATGAATTCTCGATTGATGTGG-3'	5'-GGAGGATGATAAAGATGGTGGC-3'	S3
Kng2	5'-AGCTGTGACCTTCATCCAGGA -3'	5'- TGACCAAGCACCTCCTTCAG -3'	-
Eef2	5'- TGTCAGTCATCGCCCATGTG-3'	5'- CATCCTTGCGAGTGTCAGTGA -3'	S4
S18	5'- AACGAACGAGAC TCTGGCAT-3'	5'- ACGCCACTTG TCCCTCTAAG-3'	S5

Disease Models & Mechanisms • Supplementary information

Table S5. Mouse urine albumin and creatinine concentrations

Available for download at
https://journals.biologists.com/dmm/article-lookup/doi/10.1242/dmm.052361#supplementary-data

Table S6. Mouse serum cytokine concentrations

Available for download at
https://journals.biologists.com/dmm/article-lookup/doi/10.1242/dmm.052361#supplementary-data

Table S7. Kidney tissue RT-qPCR Ct values

Available for download at
https://journals.biologists.com/dmm/article-lookup/doi/10.1242/dmm.052361#supplementary-data

Table S8. Kidney cortex F4/80-positive area per frame

Available for download at
https://journals.biologists.com/dmm/article-lookup/doi/10.1242/dmm.052361#supplementary-data

Table S9. Glomerular Nephrin and ZO1 granularity scores

Available for download at
https://journals.biologists.com/dmm/article-lookup/doi/10.1242/dmm.052361#supplementary-data

Table S10. Glomerular vWF-positive area and Nephrin granularity scores

Available for download at
https://journals.biologists.com/dmm/article-lookup/doi/10.1242/dmm.052361#supplementary-data

Table S11. Glomerular TEM image frames with quantified vesicle diameter

Available for download at
https://journals.biologists.com/dmm/article-lookup/doi/10.1242/dmm.052361#supplementary-data

Supplementary References

S1. Nakano D, Kitada K, Wan N, et al. Lipopolysaccharide induces filtrate leakage from renal tubular lumina into the interstitial space via a proximal tubular Toll-like receptor 4-dependent pathway and limits sensitivity to fluid therapy in mice. *Kidney Int*. 2020;97(5):904-912. doi:10.1016/j.kint.2019.11.024

S2. Salmenkari H, Pasanen L, Linden J, Korpela R, Vapaatalo H. Beneficial anti-inflammatory effect of angiotensin-converting enzyme inhibitor and angiotensin receptor blocker in the treatment of dextran sulfate sodium-induced colitis in mice. *J Physiol Pharmacol Off J Pol Physiol Soc*. 2018;69(4). doi:10.26402/jpp.2018.4.07

S3. Ehara H, Tatsumi K, Takafuji Y, et al. Role of tissue factor in delayed bone repair induced by diabetic state in mice. *PLOS ONE*. 2021;16(12):e0260754. doi:10.1371/journal.pone.0260754

S4. Eissa N, Hussein H, Wang H, Rabbi MF, Bernstein CN, Ghia JE. Stability of Reference Genes for Messenger RNA Quantification by Real-Time PCR in Mouse Dextran Sodium Sulfate Experimental Colitis. *PloS One*. 2016;11(5):e0156289. doi:10.1371/journal.pone.0156289

S5. Salmenkari H, Holappa M, Forsgard RA, Korpela R, Vapaatalo H. Orally administered angiotensin-converting enzyme-inhibitors captopril and isoleucine-proline-proline have distinct effects on local renin-angiotensin system and corticosterone synthesis in dextran sulfate sodium-induced colitis in mice. *J Physiol Pharmacol Off J Pol Physiol Soc*. 2017;68(3):355-362.

Disease Models & Mechanisms • Supplementary information
